# Optogenetic Manipulations of Amygdala Neurons Modulate Spinal Nociceptive Processing and Behavior Under Normal Conditions and in an Arthritis Pain Model

**DOI:** 10.3389/fphar.2021.668337

**Published:** 2021-05-25

**Authors:** Mariacristina Mazzitelli, Kendall Marshall, Andrew Pham, Guangchen Ji, Volker Neugebauer

**Affiliations:** ^1^ Department of Pharmacology and Neuroscience, School of Medicine, Texas Tech University Health Sciences Center, Lubbock, TX, United States; ^2^ Center of Excellence for Translational Neuroscience and Therapeutics, Texas Tech University Health Sciences Center, Lubbock, TX, United States; ^3^ Garrison Institute on Aging, Texas Tech University Health Sciences Center, Lubbock, TX, United States

**Keywords:** amygdala, spinal dorsal horn, pain modulation, optogenetics, electrophysiology, corticotropin releasing hormone, corticotropin releasing factor

## Abstract

The amygdala is an important neural substrate for the emotional–affective dimension of pain and modulation of pain. The central nucleus (CeA) serves major amygdala output functions and receives nociceptive and affected–related information from the spino-parabrachial and lateral–basolateral amygdala (LA–BLA) networks. The CeA is a major site of extra–hypothalamic expression of corticotropin releasing factor (CRF, also known as corticotropin releasing hormone, CRH), and amygdala CRF neurons form widespread projections to target regions involved in behavioral and descending pain modulation. Here we explored the effects of modulating amygdala neurons on nociceptive processing in the spinal cord and on pain-like behaviors, using optogenetic activation or silencing of BLA to CeA projections and CeA–CRF neurons under normal conditions and in an acute pain model. Extracellular single unit recordings were made from spinal dorsal horn wide dynamic range (WDR) neurons, which respond more strongly to noxious than innocuous mechanical stimuli, in normal and arthritic adult rats (5–6 h postinduction of a kaolin/carrageenan–monoarthritis in the left knee). For optogenetic activation or silencing of CRF neurons, a Cre–inducible viral vector (DIO–AAV) encoding channelrhodopsin 2 (ChR2) or enhanced Natronomonas pharaonis halorhodopsin (eNpHR_3.0_) was injected stereotaxically into the right CeA of transgenic Crh–Cre rats. For optogenetic activation or silencing of BLA axon terminals in the CeA, a viral vector (AAV) encoding ChR2 or eNpHR_3.0_ under the control of the CaMKII promoter was injected stereotaxically into the right BLA of Sprague–Dawley rats. For wireless optical stimulation of ChR2 or eNpHR_3.0_ expressing CeA–CRF neurons or BLA–CeA axon terminals, an LED optic fiber was stereotaxically implanted into the right CeA. Optical activation of CeA–CRF neurons or of BLA axon terminals in the CeA increased the evoked responses of spinal WDR neurons and induced pain-like behaviors (hypersensitivity and vocalizations) under normal condition. Conversely, optical silencing of CeA–CRF neurons or of BLA axon terminals in the CeA decreased the evoked responses of spinal WDR neurons and vocalizations, but not hypersensitivity, in the arthritis pain model. These findings suggest that the amygdala can drive the activity of spinal cord neurons and pain-like behaviors under normal conditions and in a pain model.

## Highlights

• Optical activation of BLA axon terminals in CeA or of CeA–CRF neurons increased the evoked activity of spinal dorsal horn neurons and induced nocifensive and emotional responses under normal conditions, mimicking the pain state.
• Optical silencing of BLA axons in CeA or of CeA–CRF neurons in an arthritis pain model decreased the enhanced activity of spinal dorsal horn neurons and reduced emotional responses, but not the hypersensitivity.
• The data provide direct evidence for the modulation of pain-like behaviors and spinal neuronal activity by BLA–CeA signaling and CeA–CRF neurons on under normal conditions and in an arthritis pain model.

## Introduction

Pain is a complex medical condition resulting from the mutual interaction of multiple components, such as sensory, cognitive and emotional–affective. The amygdala, a limbic brain structure, plays a critical role in the affective aspects of behavior and in pain modulation (Veinante et al., 2013; Vachon-Presseau et al., 2016a; Kato et al., 2018; Neugebauer, 2020). The amygdala consists of distinct nuclei, including the central nucleus (CeA), the basolateral complex (BLA), and interposed between them the intercalated cell clusters (ITC). The CeA serves major amygdala output functions and receives purely nociceptive information from the spinal cord via the parabrachial (PB) area of the brainstem and highly integrated multimodal inputs from the thalamus and cortex via the BLA complex (Veinante et al., 2013; Kato et al., 2018; Thompson and Neugebauer, 2019; Neugebauer, 2020). The amygdala processes information from different brain regions and connects to ascending and descending pain modulatory systems and other areas of the central nervous system (CNS) involved in behaviors and cognitive and emotional functions (DeBerry et al., 2015).

The BLA is composed of glutamatergic pyramidal neurons that project to the CeA as well as to several cortical regions, including the medial prefrontal cortex (mPFC) and anterior cingulate cortex (ACC) (Neugebauer et al., 2009; Toyoda et al., 2011; Veinante et al., 2013; Janak and Tye, 2015; Neugebauer, 2020). Through associative processing, emotional-affective significance is conferred to polymodal sensory inputs from the thalamus and cortex that are transmitted to the CeA for further processing. The BLA–CeA circuit has been implicated in the generation and modulation of pain-like behaviors (Thompson and Neugebauer, 2017; Corder et al., 2019; Neugebauer, 2020). The CeA is mainly composed by GABAergic neurons and many co-express neuropeptides such as somatostatin (SOM), protein kinase C delta (PKCδ), dynorphin and corticotropin releasing factor (CRF). Interestingly, expression of those neuropeptides signifies distinct neuronal CeA subpopulations that may serve different functions in anxiety, fear and pain (Haubensak et al., 2010; Li et al., 2013; McCullough et al., 2018; Wilson et al., 2019; Neugebauer et al., 2020). Importantly, the CeA contains the highest density of extra–hypothalamic CRF neurons, which project to different areas of the CNS important for regulating behaviors and pain (Pomrenze et al., 2015). CRF cell bodies are found mostly in the lateral division (CeL) of the CeA and their projections target several nuclei of the hypothalamus, the bed nucleus of the stria terminalis and different areas of the brainstem, including periaqueductal gray (PAG) and PB (Pomrenze et al., 2015; De Guglielmo et al., 2019). CeA–CRF neurons have been linked to the pain modulatory effects of kappa opioid receptors (Ji and Neugebauer, 2020; Hein et al., 2021).

Here we test the hypothesis that selective activation of BLA–CeA terminals or CeA–CRF neurons has facilitatory effects under normal condition and selective silencing of these elements has beneficial inhibitory effects in a pain state. To address our hypothesis, we combined optogenetic strategies with electrophysiological and behavioral assays. Optogenetics utilize genetically–modified light–sensitive channels (opsins) to target specific neuronal populations, allowing the control of neuronal activity with light (Deisseroth, 2015).

## Results

**FIGURE 1:**
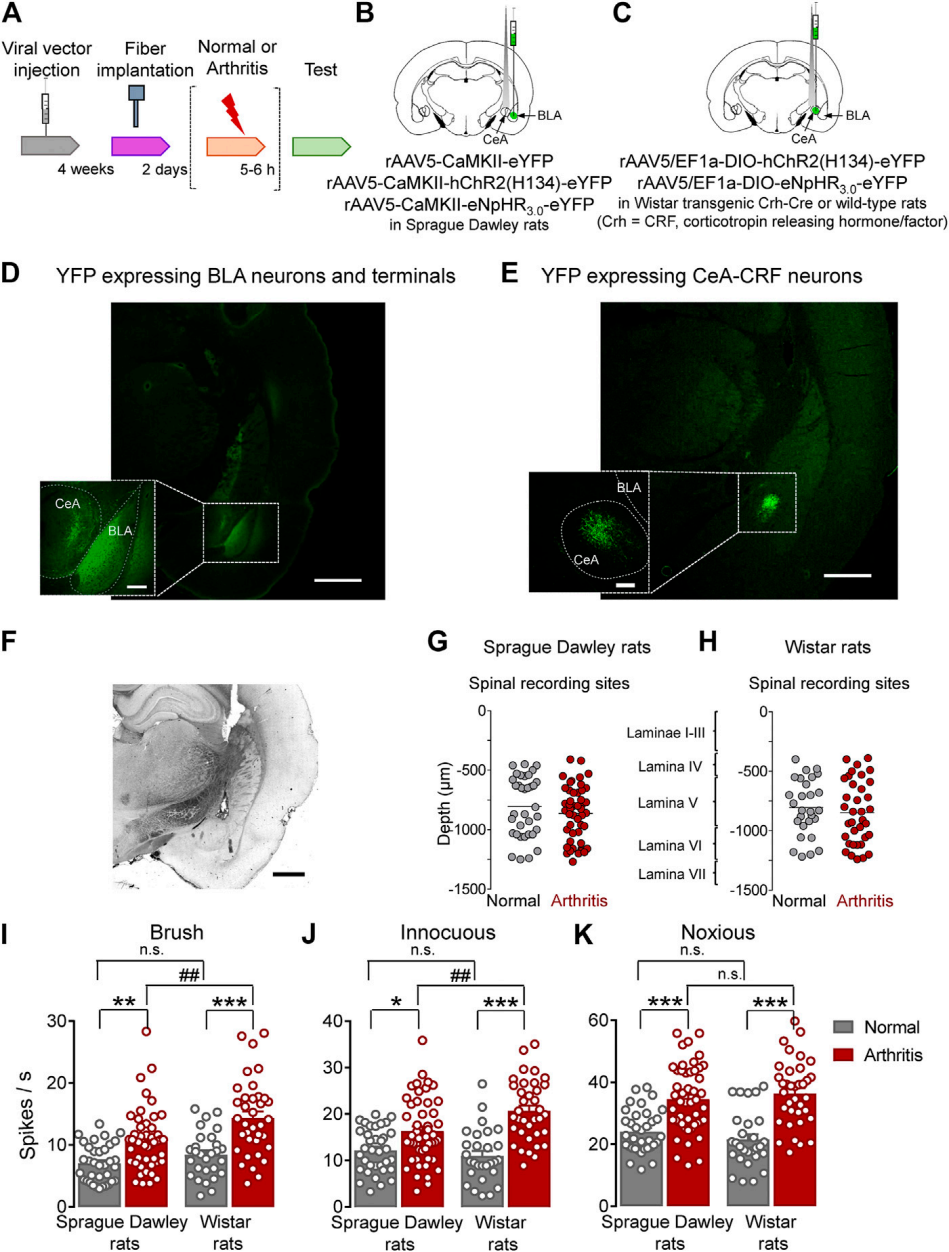
Overview of experimental strategy and arthritis pain-related changes in spinal dorsal horn neurons. **(A)** Experimental design. **(B)** Schematic illustration of viral vector injection into the BLA to express light–sensitive channels under the control of CaMKII in BLA–CeA axon terminals, and application of light into CeA. **(C)** Schematic illustration of the injection of the Cre-inducible viral vector into the CeA to express light–sensitive channels in CRF neurons, and application of light into CeA. **(D)** Representative image of viral vector–mediated YFP expression in BLA and YFP expressing BLA axon terminals in CeA (green, eYFP). Scale bars, 1000 µm (lower magnification image), 200 µm (higher magnification image). **(E)** Representative image of viral vector-mediated YFP expression in CeA–CRF neurons (green, eYFP). Scale bars, 1000 µm (lower magnification image), 200 µm (higher magnification image). **(F)** LED fiber track into the CeA. Scale bar 1000 µm **(G)** Depths of recording electrode tips from the dorsal surface of the spinal cord of Sprague Dawley rats. **(H)** Depths of recording electrodes in the spinal cord of Wistar rats. Also shown is the estimated correlation with dorsal horn laminae. **(I–K)** Spinal neuronal activity evoked by brushing the skin **(I)** and innocuous **(J)** and noxious **(K)** compression of the left knee joint was increased in arthritic SD and arthritic Wistar rats compared to normal naïve rats. In normal naïve rats, no significant difference was found in evoked responses between SD and Wistar strains. In the arthritis pain model, neuronal activity evoked by innocuous **(I, J)**, but not noxious **(K)**, stimulation of the arthritic knee was significantly higher in arthritic Wistar rats than arthritic SD rats. Bar histograms show means ± SEM. SD: Normal, *n* = 35 in 13 rats; Arthritis, *n* = 48 in 18 rats; Wistar: Normal, *n* = 28 in 12 rats; Arthritis, *n* = 36 in 14 rats. *, **, ***p < 0.05, 0.01, 0.001 compared to normal; *p* < 0.01 compared to SD; one–way ANOVA with Bonferroni posthoc tests.

This study investigated the contribution of optical manipulation of BLA axon terminals in the CeA and of CeA–CRF neurons on the neuronal activity of spinal dorsal horn neurons and pain–like behaviors under normal conditions and in an arthritis pain model (*Arthritis pain model*). To do so, viral vectors coding light–sensitive channels were injected into BLA or CeA, and an optical fiber delivering light of appropriate length was implanted 4 weeks after surgery and 2 days before testing (Figure 1A). For optical manipulation of BLA axon terminals in the CeA, we stereotaxically injected adeno–associated viral vector encoding channelrhodopsin 2 (ChR2) or enhanced Natronomonas pharaonis halorhodopsin (eNpHR_3.0_) fused to yellow fluorescent protein (YFP) under the control of CaMKII promoter into the BLA of SD rats (Figure 1B). For control experiments, only YFP was expressed in the BLA. For optical manipulation of CRF neurons in the CeA, we stereotaxically injected a Cre–inducible (DIO) adeno–associated viral vector encoding ChR2 or enhanced eNpHR_3.0_ fused to YFP into the CeA of transgenic Crh–Cre rats (Crh = CRF, corticotropin releasing hormone/factor) (Figure 1C). For control experiments, a viral vector was injected into the CeA of wild type rats. Histological verification confirmed selective eYFP expression in BLA or CeA (Figures 1D,E). An LED fiber was stereotaxically inserted into the CeA to deliver blue (473 nm) light for activation or yellow (590 nm) light for inhibition (Figure 1F).

Extracellular single unit recordings from WDR neurons in the deep dorsal horn (lamina IV–VI; mostly lamina V) of the lumbar enlargement (L2–L4, Figures 1G,H) showed significantly increased responses to innocuous brushing the skin (I) and to innocuous (J) and to noxious (K) compression of the arthritic (left) knee with a calibrated forceps (10 s, *In vivo spinal cord electrophysiology*) in arthritic compared to normal naïve SD rats (Normal, *n* = 35 neurons in 13 rats; Arthritis, *n* = 48 in 18 rats; Figure 1I, *p* < 0.01, F_(3, 143)_ = 16.53; Figure 1J, *p* < 0.05, F_(3, 143)_ = 16.75; Figure 1K, *p* < 0.001, F_(3, 143)_ = 20.88; one-way ANOVA with Bonferroni posthoc tests) and in arthritic compared to normal naïve Wistar rats (Normal, *n* = 28 neurons in 12 rats; Arthritis, *n* = 36 in 14 rats; Figure 1I, *p* < 0.001; Figure 1J, *p* < 0.001; Figure 1K, *p* < 0.001; for F values see above, one–way ANOVA with Bonferroni posthoc tests). The responses to the three outcome measures were not significantly different between SD and Wistar strains under normal conditions (*p* > 0.05, one–way ANOVA with Bonferroni posthoc tests). While qualitatively similar increases in the arthritis pain model were found for SD and Wistar rats, the spinal neuronal activity evoked by innocuous brushing the skin and innocuous, but not noxious, compression of the arthritic knee was significantly higher in arthritic Wistar than arthritic SD rats (Figure 1I, *p* < 0.01; Figure 1J, *p* < 0.0; Figure 1K, *p* > 0.05; for F values see above, one–way ANOVA with Bonferroni posthoc tests).

### Facilitatory Effects of Optical Activation of BLA–CeA Terminals on Spinal Nociceptive Activity in Naïve Rats

**FIGURE 2:**
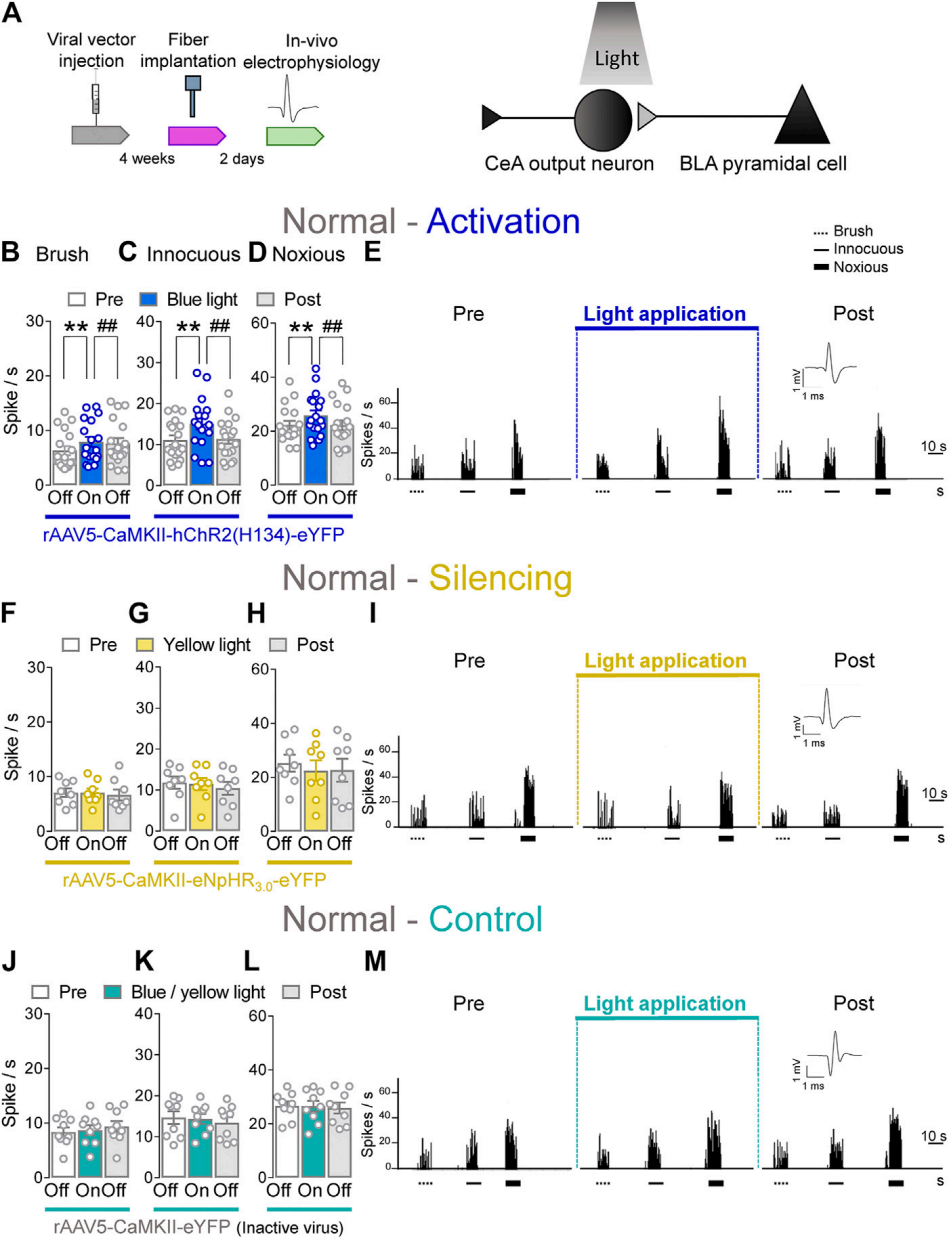
Optogenetic activation of BLA axon terminals in the CeA under normal condition increases evoked activity of spinal cord neurons. **(A)** Experimental design. Neuronal WDR activity (*n* = 18) evoked by brushing the skin **(B)** and innocuous **(C)** and noxious **(D)** compression of the left knee joint was significantly enhanced by optical activation of ChR2 on the BLA–CeA terminals with blue (473 nm) light (“On”, 20 Hz, 5 mW, 5–10 min) compared to no light (“Off”) values in normal naive rats. **(E)** Peristimulus time histograms (500 ms bin size) show recordings of an individual WDR neuron before, during and after blue (473 nm) light application (20 Hz, 5 mW, 5–10 min) in a normal naïve rat. Bar histograms show means ± SEM. **, ^##^ *p* < 0.01, repeated measures one-way ANOVA with Bonferroni posthoc tests **(F–H)** Same display as **(B–D)** but for BLA–CeA terminal inhibition with yellow (590 nm) light (“On”) to activate eNpHR_3.0_ (*n* = 8). **(I)** Same display as **(E)** but for BLA–CeA terminal silencing. **(J–L)** Same display as **(B–D)** but in YFP–expressing control rats with either blue (473 nm, *n* = 4) or yellow (590 nm, *n* = 5) light (“On”) application (*n* = 9). **(M)** Same display as **(E)** but in a YFP–expressing control rat.

To determine the contribution of BLA–CeA terminals on spinal dorsal horn neurons, extracellular single unit recordings of WDR neurons in the deep layers of the spinal dorsal horn (L2–L4) were performed 4 weeks after rAAV5–CaMKII–(opsin)–eYFP or rAAV5–CaMKII–eYFP (control) injections into the BLA in normal rats. Optical stimulation of ChR2 or eNpHR_3.0_ was achieved by the stereotaxic implantation of an LED optical fiber for blue (473 nm) or yellow (590 nm) light into the CeA 2 days before testing (Figure 2A, *Optogenetic strategy* and *In vivo spinal cord electrophysiology*). Activation of ChR2 at the BLA–CeA terminals by blue (473 nm) light (“On”, 5–10 min, 20 Hz, 5 mW) significantly increased the activity of WDR neurons evoked by brief (10 s) innocuous brush (*p* < 0.01, F_(2, 34)_ = 12.1) and innocuous (*p* < 0.01, F_(2, 34)_ = 11.89) and noxious compression (*p* < 0.01, F_(2, 34)_ = 7.153; repeated measures one–way ANOVA with Bonferroni posthoc tests, *n* = 18) of the left knee joint with a calibrated forceps (*In vivo spinal cord electrophysiology*) compared to no light exposure (“Off”) in normal rats (Figures 2B–E). The effects of optical activation were reversible (Figures 2B–E).

Conversely, optogenetic silencing of eNpHR_3.0_ expressing BLA–CeA terminals by yellow (590 nm) light (“On”, 5–10 min, 20 Hz, 5 mW) application had no significant effects on the responses of WDR neurons to brief (10 s) brushing the skin (*p* > 0.05, F_(2, 14)_ = 0.5215) and innocuous (*p* > 0.05, F_(2, 14)_ = 1.64) and noxious compression (*p* > 0.05, F_(2, 14)_ = 0.9986, repeated measures one–way ANOVA with Bonferroni posthoc tests, *n* = 8) of the left knee joint compared to no light application (“Off”) in normal rats (Figures 2F–I). In YFP–control rats, the application of blue (473 nm) or yellow (590 nm) light (“On”, 5–10 min, 20 Hz, 5 mW) into the CeA did not affect the evoked responses of spinal WDR neurons (*p* > 0.05; Figure 2J, F_(2, 16)_ = 1.982; Figure 2K, F_(2, 16)_ = 1.757; Figure 2L, F_(2, 16)_ = 0.1571; repeated measures one–way ANOVA with Bonferroni posthoc tests, *n* = 9) compared to no light exposure (“Off”) in normal rats (Figures 2J–M). Data for blue (*n* = 4) and yellow (*n* = 5) light exposure in control rats were combined because no difference was observed. Optical manipulations in the amygdala did not induce any non-evoked (by peripheral stimuli) activity in spinal neurons. The data suggest that activation of BLA–CeA inputs has facilitatory effects on spinal nociceptive processing under normal condition.

Whole–cell patch clamp experiments (*Patch–clamp electrophysiology in amygdala slices*) were performed to validate our optogenetics approach (Supplementary Figure S1). Application of continuous or pulsed (20 Hz) blue light for 5 s at the BLA–CeA terminals expressing ChR2 (Supplementary Figure S1A) induced firing activity of a CeA neuron in the laterocapsular division (CeLC) in an amygdala–containing slice obtained from a normal rat. No difference was detected between continuous and pulsed light exposure on the CeLC neuronal activity, validating our experimental approach using pulsed light exposure in the CeA in the *in vivo* studies. Yellow light was applied as control (Supplementary Figures S1B, C).

### Inhibitory Effects of Optical Silencing of BLA–CeA Terminals on Spinal Nociceptive Activity in Arthritic Rats

**FIGURE 3:**
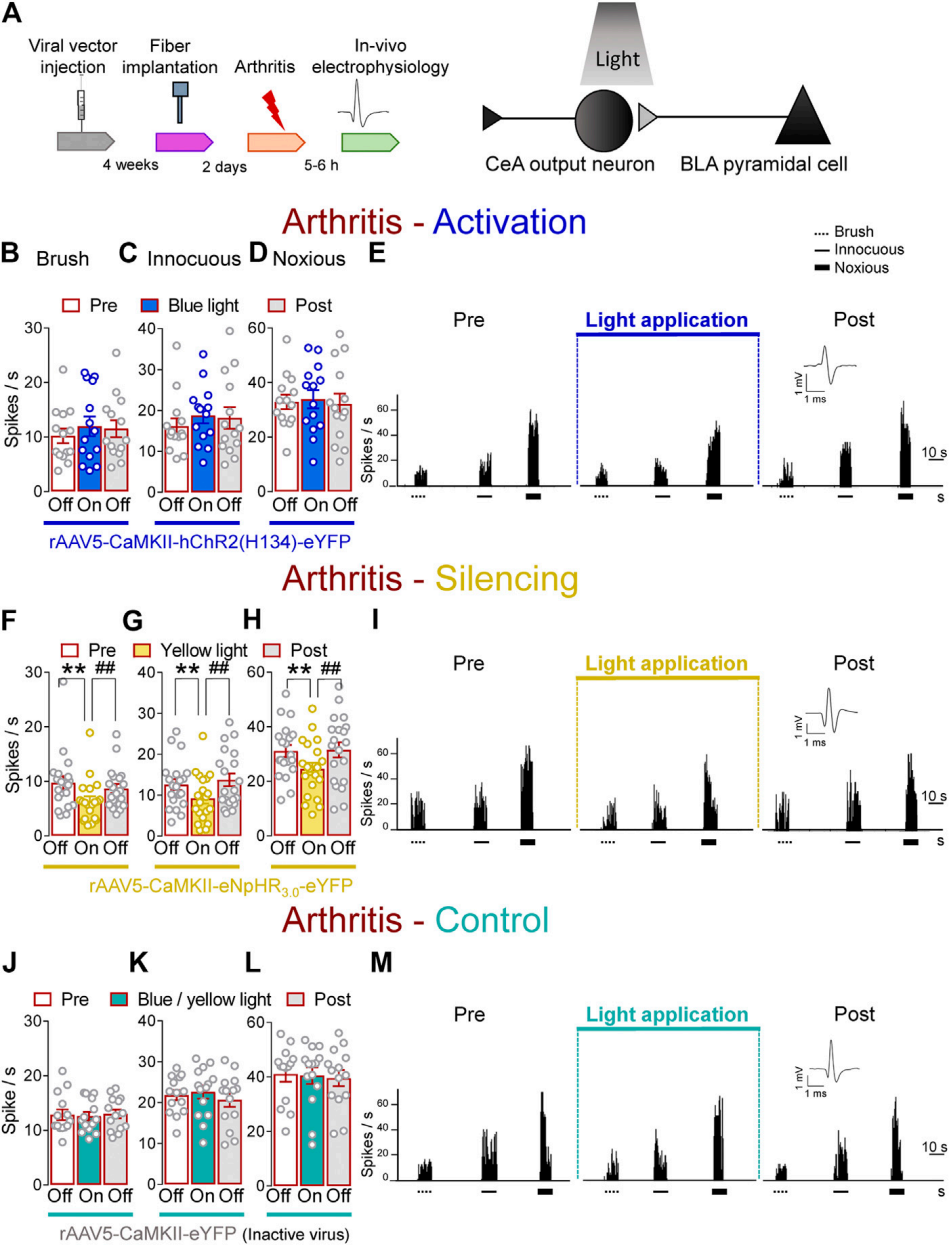
Optogenetic silencing of BLA axon terminals in the CeA in the arthritis pain model reduces the increased activity of spinal cord neurons. **(A)** Experimental design. Responses of WDR neurons (*n* = 14) to brushing the skin **(B)** and innocuous **(C)** and noxious **(D)** compression of the left knee joint were not significantly affected by the optical activation of ChR2 expressed on BLA–CeA terminals with blue (473 nm) light (“On”, 20 Hz, 5 mW, 5–10 min) compared to no light (“Off”) values in arthritic rats. **(E)** Peristimulus time histograms (500 ms bin size) show recordings of a single WDR neuron before, during and after blue (473 nm) light application (20 Hz, 5 mW, 5–10 min) in an arthritic rat. Bar histograms show means ± SEM . **(F–H)** Same display as **(B–D)** but for BLA–CeA terminal inhibition with yellow (590 nm) light (“On”) to activate eNpHR_3.0_ (*n* = 8). **(I)** Same display as **(E)** but for BLA–CeA terminal silencing (*n* = 20). **, ^##^ *p* < 0.01, repeated measures one–way ANOVA with Bonferroni posthoc tests. **(J–L)** Same display as **(B–D)** but in YFP–expressing control rats with blue (473 nm, *n* = 7) or yellow (590 nm, *n* = 7) light (“On”) application (*n* = 14). **(M)** Same display as **(E)** but in a YFP–expressing control rat.

Next, we investigated the contribution of optical manipulations of the BLA–CeA terminals to spinal nociceptive processing in the arthritis pain model (Figure 3A). The evoked responses of dorsal horn WDR neurons were enhanced in arthritic rats (5–6 h post induction) compared to normal conditions (Figures 1I–K). In the arthritis condition, activation of BLA–CeA terminals by blue (473 nm) light (“On”, 5–10 min, 20 Hz, 5 mW) delivered through an optical fiber stereotaxically implanted into the CeA (*Optogenetic strategy*) had no significant effect on the responses of WDR neurons to brief (10 s) brushing the skin (*p* > 0.05, F_(2, 26)_ = 1.642) and innocuous (*p* > 0.05, F_(2, 26)_ = 1.36) and noxious compression (F_(2, 26)_ = 0.2889, repeated measures one–way ANOVA with Bonferroni posthoc tests, *n* = 14) of the left knee joint with a calibrated forceps (*In vivo spinal cord electrophysiology*) compared to no light exposure (“Off”, Figures 3B–E).

In contrast, optogenetic silencing of BLA terminals in the CeA by yellow (590 nm) light (“On”, 5–10 min, 20 Hz, 5 mW) application into the CeA, had significant inhibitory effects on the activity of WDR neurons evoked by brush (*p* < 0.01, F_(2, 38)_ = 20.94) and innocuous (*p* < 0.01, F_(2, 38)_ = 10.54) and noxious (*p* < 0.01, F_(2, 38)_ = 18.53, repeated measures one–way ANOVA with Bonferroni posthoc tests, *n* = 20) stimulation of the left (arthritic) knee joint compared to no light exposure (“Off”) in arthritic rats (Figures 3F–I). The effects of optical inhibition were reversible (Figures 3F–I). In YFP–control rats, application of blue (473 nm) or yellow (590 nm) light (“On”, 5–10 min, 20 Hz, 5 mW) delivered by an optical fiber in the CeA (*Optogenetic strategy*) did not affect the evoked responses of WDR neurons by brief (10 s) mechanical stimulation (*p* > 0.05; Figures 3J, F_(2, 26)_ = 0.3145; Figures 3K, F_(2, 26)_ = 3.092; Figures 3L, F_(2, 26)_ = 0.382; repeated measures one–way ANOVA with Bonferroni posthoc tests, *n* = 14) of the knee joint compared to no light application (“Off”) in arthritic rats (Figures 3J–M). Data for blue (*n* = 7) and yellow (*n* = 7) light exposure were combined because no difference was detected. These results suggest inhibitory effects of silencing BLA–CeA terminals on spinal nociceptive processing in an arthritis pain model.

Brain slice physiology showed that application of continued or pulsed (20 Hz) yellow light for 5 s to the BLA–CeA terminals expressing eNpHR_3.0_ inhibited action potential firing of CeLC neuron in a brain slice from an arthritic rat. No difference was detected between the effects of continuous and pulsed light exposure on CeLC neuronal activity, supporting our experimental approach using pulsed light in the *in vivo* studies. Blue light was applied as control (Supplementary Figures S1D, E).

### Facilitatory Effects of Optical Activation of CeA–CRF Neurons on Spinal Nociceptive Activity in Naïve Rats

**FIGURE 4:**
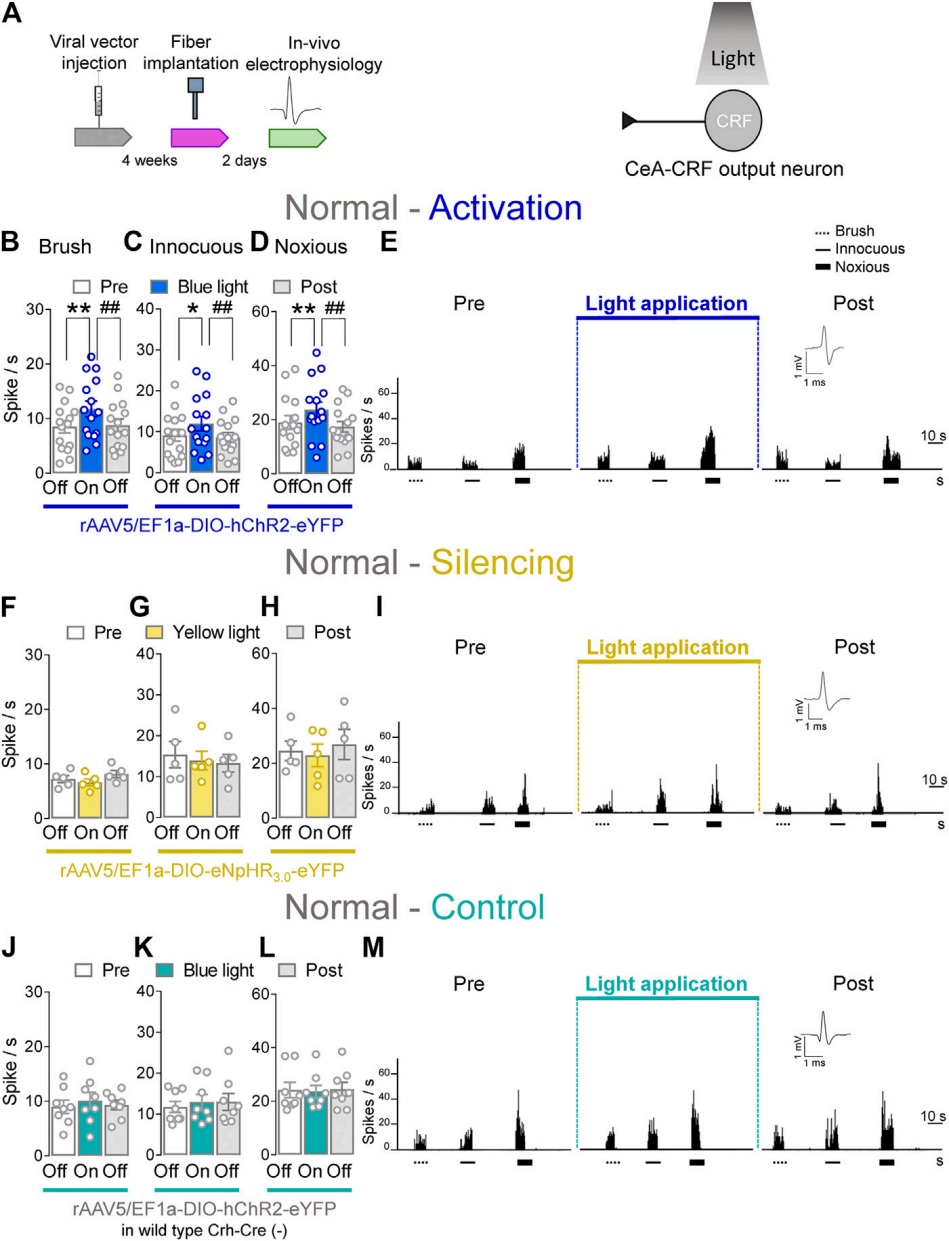
Optogenetic activation of CeA-CRF neurons under normal conditions increases evoked activity of spinal cord neurons. **(A)** Experimental design. Responses of WDR neurons (*n* = 15) to brushing the skin **(B)** and innocuous **(C)** and noxious **(D)** compression of the left knee joint were significantly enhanced by optical activation of ChR2 expressing CeA–CRF neurons with blue (473 nm) light (“On”, 20 Hz, 5 mW, 5–10 min) compared to no light (“Off”) values in normal naive rats. **(E)** Peristimulus time histograms (500 ms bin size) show recordings of an individual WDR neuron recorded before, during and after blue (473 nm) light application (20 Hz, 5 mW, 5–10 min) in a normal naïve rat. Bar histograms show means ± SEM. *, *p* < 0.05, **, ^##^ *p* < 0.01, repeated measures one–way ANOVA with Bonferroni posthoc tests. **(F–H)** Same display as **(B–D)** but for silencing of eNpHR_3.0_ expressing CeA–CRF neurons with yellow (590 nm) light (“On”) exposure (*n* = 5). (I) Same display as **(E)** but for CeA–CRF neuron silencing. **(J–L)** Same display as **(B–D)** but in wild type control rats with blue (473 nm) light (“On”) application (*n* = 8). **(M)** Same display as **(E)** but in a wild type control rat.

CeA–CRF neurons are involved in behavioral modulation, pain and fear (Pomrenze et al., 2015; Neugebauer et al., 2020). Therefore, we aimed to explore the involvement of CeA–CRF neurons on the activity of spinal neurons using optogenetics in transgenic Crh–Cre rats. Extracellular single unit recording from WDR neurons in the deep dorsal horn (L2–L4) were performed 4 weeks after rAAV5–DIO–(opsin)–eYFP injections into the CeA in normal rats to express ChR2 or eNpHR_3.0_ in CeA–CRF neurons. An LED optical fiber delivering blue (473 nm) or yellow (590 nm) light was stereotaxically implanted into the CeA 2 days before testing to stimulate ChR2 or eNpHR3.0 (Figure 4A, *Optogenetic strategy* and *In vivo spinal cord electrophysiology*). Activation of CeA–CRF neurons by blue (473 nm) light (“On”, 5–10 min, 20 Hz, 5 mW) significantly increased the activity of WDR neurons evoked by brief (10 s) innocuous brush (*p* < 0.01, F_(2, 28)_ = 13.44) and innocuous (*p* < 0.05, F_(2, 28)_ = 7.622) and noxious mechanical stimulation (*p* < 0.01, F_(2, 28)_ = 10.04; repeated measures one–way ANOVA with Bonferroni posthoc tests, *n* = 15) of the left knee joint with a calibrated forceps (*In vivo spinal cord electrophysiology*) compared to no light application (“Off”) under normal condition (Figures 4B–E). The effects of optical activation were reversible (Figures 4B–E).

Conversely, optical silencing of CeA–CRF neurons by yellow (590 nm) light application (“On”, 5–10 min, 20 Hz, 5 mW) had no significant effects on the activity of WDR neurons evoked by brief (10 s) brushing the skin (*p* > 0.05, F_(2, 8)_ = 3.796) and innocuous (*p* > 0.05, F_(2, 8)_ = 0.36150) and noxious (*p* > 0.05, F_(2, 8)_ = 1.467; repeated measures one–way ANOVA with Bonferroni posthoc tests, *n* = 5) stimulation of the left knee joint compared to no light exposure (“Off”) in normal naive rats (Figures 4F–I). In normal wild type rats (control), the application of blue (473 nm) light (“On”, 5–10 min, 20 Hz, 5 mW) did not significantly change the responses of WDR neurons to mechanical stimulation of the knee joint (*p* > 0.05; Figure 4J, F_(2, 14)_ = 0.7263; Figure 4K, F_(2, 14)_ = 0.9692; Figure 4L, F_(2, 14)_ = 0.09817; repeated measures one–way ANOVA with Bonferroni posthoc tests, *n* = 8) compared to no light exposure (“Off”; Figures 4J–M). The data suggest that activation of CeA–CRF neurons has facilitatory effects on spinal cord neuronal activity under normal conditions.

Whole–cell patch clamp recordings (*Patch–clamp electrophysiology in amygdala slices* and Supplementary Figures S2A–C) were made of CeA–CRF neurons identified by their YFP expression (De Guglielmo et al., 2019). Application of continuous or pulsed (20 Hz) blue light for 5 s induced action potential firing of a CRF neuron in an amygdala slice from a normal rat. No difference was detected between continuous and pulsed light effects on CeA–CRF activity, supporting our experimental approach using pulsed light in the *in vivo* studies. Yellow light was applied as control (Supplementary Figures S2B,C).

### Inhibitory Effects of Optical Silencing of CeA–CRF Neurons on Spinal Nociceptive Activity in Arthritic Rats

**FIGURE 5:**
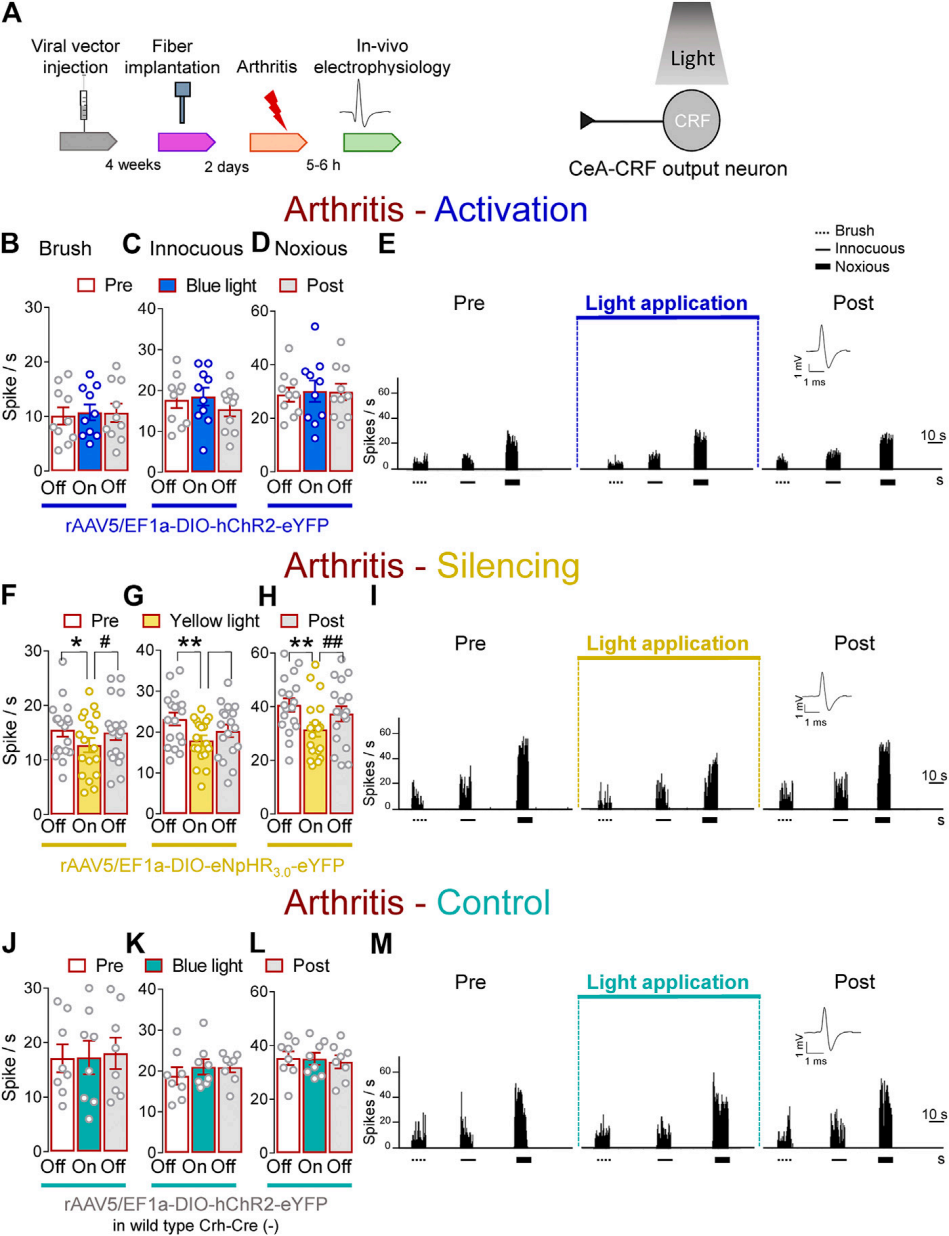
Optogenetic silencing of CeA-CRF neurons in the arthritis pain model reduces the increased activity of spinal cord neurons. **(A)** Experimental design. Responses of WDR neurons (*n* = 10) to brushing the skin **(B)** and innocuous **(C)** and noxious **(D)** compression of the left knee joint were not significantly affected by optical activation of ChR2 expressing CeA–CRF neurons with blue (473 nm) light (“On”, 20 Hz, 5 mW, 5–10 min) compared to no light (“Off”) values in arthritic rats. **(E)** Peristimulus time histograms (500 ms bin size) of a single WDR neuron recorded before, during and after blue (473 nm) light application (20 Hz, 5 mW, 5–10 min) in an arthritic rat. Bar histograms show means ± SEM. **(F–H)** Same display as **(B–D)** but for silencing of eNpHR_3.0_ expressing CeA–CRF neurons with yellow (590 nm) light (“On”). **(I)** Same display as **(E)** but for CeA–CRF neuron silencing (*n* = 18). *, **p < 0.05, 0.01 compared to no light (“Off”) values; *p* < 0.05, 0.01 compared to light (“On”) values, repeated measures one–way ANOVA with Bonferroni posthoc tests. **(J–L)** Same display as **(B–D)** but in wild type control rats with blue (473 nm) light (“On”) application (*n* = 8). **(M)** Same display as (**E)** but in a wild type control rat.

Next, we determined the effects of optical manipulations of CeA–CRF neurons on spinal nociceptive processing in the arthritis pain model (Figure 5A). The responses of spinal WDR neurons were enhanced in arthritic rats (5–6 h post induction) compared to normal conditions (Figures 1I–K). In the arthritis pain model, activation of CeA–CRF neurons by blue (473 nm) light (“On”, 5–10 min, 20 Hz, 5 mW) through an optical fiber in the CeA (*Optogenetic strategy*) had no significant effects on the responses of WDR neurons to brief (10 s) brushing the skin (*p* > 0.05, F_(2, 18)_ = 0.4702) and to innocuous (*p* > 0.05, F_(2, 18)_ = 3.283) and to noxious compression (*p* > 0.05, F_(2, 18)_ = 0.3161, repeated measures one–way ANOVA with Bonferroni posthoc tests, *n* = 10) of the left (arthritic) knee joint with a calibrated forceps (*In vivo spinal cord electrophysiology*) compared to no light exposure (“Off”, Figures 5B–E).

In contrast, optogenetic silencing of CeA–CRF neurons by yellow (590 nm) light (“On”, 5–10 min, 20 Hz, 5 mW) application into the CeA significantly inhibited the activity of WDR neurons evoked by brush (*p* < 0.05, F_(2, 34)_ = 5.383) and innocuous (*p* < 0.01, F_(2, 34)_ = 8.554) and noxious (*p* < 0.01, F_(2, 34)_ = 15.08, repeated measures one–way ANOVA with Bonferroni posthoc tests, *n* = 18) stimulation of the left knee joint compared to no light exposure (“Off”) in arthritic rats (Figures 5F–I). The effects of optical inhibition were reversible (Figures 5F–I). In normal wild type rats (control), the application of blue (473 nm) light (“On”, 5–10 min, 20 Hz, 5 mW) did not change the responses of WDR neurons to brief (10 s) mechanical stimulation (*p* > 0.05; Figure 5J, F_(2, 14)_ = 0.206; Figure 5K, F_(2, 14)_ = 1.025; Figure 5L, F_(2, 14)_ = 0.1829, noxious; repeated measures one–way ANOVA with Bonferroni posthoc tests, *n* = 8) compared to no light application (“Off”) in arthritic rats (Figures 5J–M). The results suggest inhibitory effects of silencing CeA–CRF neurons on spinal nociceptive processing in an arthritis pain model.

As a validation of the optogenetic approach, application of continuous or pulsed (20 Hz) yellow light for 5 s inhibited action potential firing in a CRF neuron (*Facilitatory effects of optical activation of CeA–CRF neurons on spinal nociceptive activity*) in an amygdala slice from an arthritic rat (*Patch–clamp electrophysiology in amygdala slices* and Supplementary Figures S2D,E). No difference was detected between the effects of continuous and pulsed light exposure on CRF neuronal activity, supporting our experimental approach using pulsed light in the CeA in the *in vivo* studies. Blue light was applied as control (Supplementary Figures S2D,E).

### Effects of Optical Manipulation of Amygdala Activity on Pain–like Behaviors

**FIGURE 6:**
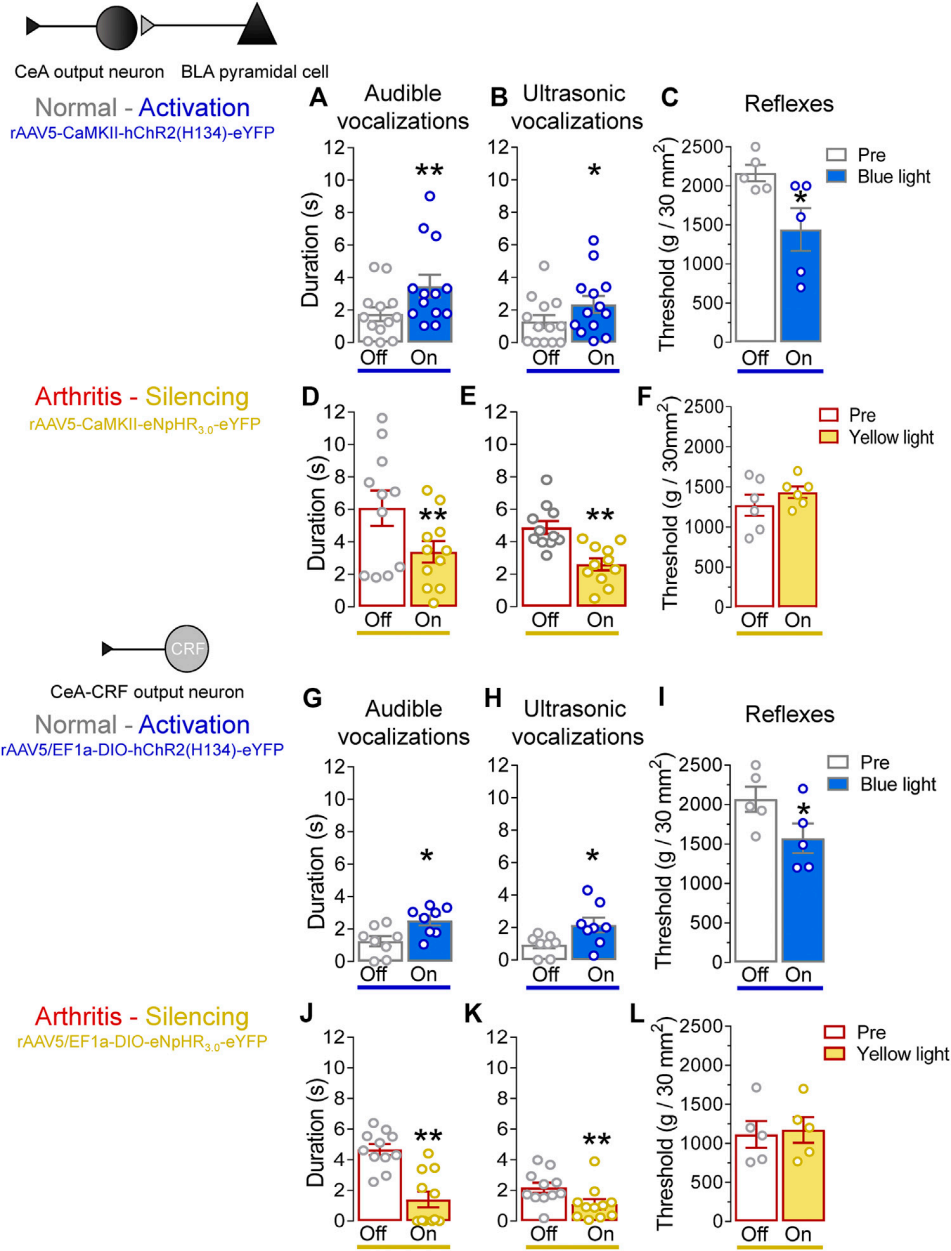
Effects of optogenetic amygdala manipulations on pain-related behaviors in normal and arthritis conditions. **(A)** Audible and **(B)** ultrasonic vocalizations evoked by noxious compression of the knee joint with a calibrated forceps were significantly increased by the activation of ChR2 on BLA–CeA terminals with blue (473 nm) light application (“On”, 20 Hz, 5 mW, 5–10 min) into the CeA compared to no light (“Off”) values in normal naïve rats (*n* = 13). **(C)** Mechanical withdrawal thresholds were significantly reduced by blue light (473 nm) exposure in normal naïve rats (*n* = 5), indicating hypersensitivity. **(D–F)** Same display as **(A–C)** but for silencing of eNpHR_3.0_ expressing BLA–CeA terminals with yellow light (590 nm) in arthritic rats (audible and ultrasonic vocalizations, *n* = 11; reflexes, *n* = 6). **(G–I)** Same display as **(A–C)** but for activation of ChR2 expressing CeA–CRF neurons in normal naïve transgenic rats (audible and ultrasonic vocalizations, *n* = 8; reflexes, *n* = 5). **(J–L)** Same display as **(A–C)** but for silencing of eNpHR_3.0_ expressing CeA–CRF neurons with yellow light (590 nm) in arthritic transgenic rats (audible and ultrasonic vocalizations, *n* = 11; reflexes, *n* = 5). *, **p < 0.05, 0.01 compared to no light (“Off”) values, paired *t*–test.

We evaluated the behavioral consequences of optogenetic manipulations of BLA–CeA terminals or CeA–CRF neurons (Figure 6, *Pain–related behaviors*) in normal and arthritic rats. Optogenetic activation of ChR2 expressing BLA–CeA terminals with blue (473 nm) light (“On”, 5–10 min, 20 Hz, 5 mW) significantly increased audible and ultrasonic vocalizations in response to brief (10 s) noxious compression of the left knee joint with a calibrated forceps (*Pain–related behaviors*) compared to no light (“Off”) in normal naïve rats (Figure 6A, *p* < 0.01, *t* = 3.533; Figure 6B, *p* < 0.05, *t* = 2.862; *n* = 13, paired *t*-test). Similarly, withdrawal thresholds of nocifensive reflexes evoked by mechanical compression of the knee joint (*Pain–related behaviors*) were significantly decreased by optical activation of BLA–CeA terminals in normal naïve rats (Figure 6C, *p* < 0.05, *t* = 3.913, *n* = 5, paired *t*-test). In the arthritis pain model, optogenetic silencing of eNpHR_3.0_ expressing BLA–CeA terminals with yellow (593 nm) light (“On”, 5–10 min, 20 Hz, 5 mW) had significant inhibitory effects on audible and ultrasonic vocalizations evoked by noxious compression of the left (arthritic) knee joint (Figure 6D, *p* < 0.01, *t* = 4.415; Figure 6E, *p* < 0.005, *t* = 4.89; *n* = 11, paired *t*-test compared to no light (“Off”)). Surprisingly, mechanical withdrawal thresholds were not affected by optical silencing of the BLA–CeA terminals in arthritic rats (Figure 6F, *p* > 0.05, *t* = 1.881, *n* = 6, paired *t*-test).

Activation of ChR2 expressing CeA–CRF neurons by the application of blue (473 nm) light (“On”, 5–10 min, 20 Hz, 5 mW) significantly increased audible and ultrasonic vocalizations evoked by brief (10 s) noxious stimulation of the left knee joint with a calibrated forceps (*Pain–related behaviors*) compared to no light (“Off”) in normal naïve transgenic rats (*p* < 0.05; Figure 6G, *t* = 3.271; Figure 6H, *t* = 2.378; *n* = 8, paired *t*-test). Optical activation of CeA–CRF neurons significantly decreased withdrawal thresholds of nocifensive reflexes evoked by mechanical compression of the knee joint (*Pain–related behaviors*) in normal naïve transgenic rats (Figure 6I, *p* < 0.05, *t* = 3.994, *n* = 5, paired *t*-test). In arthritic transgenic rats, optical silencing of eNpHR_3.0_ expressing CeA–CRF neurons with yellow (593 nm) light (“On”, 5–10 min, 20 Hz, 5 mW) significantly reduced audible and ultrasonic vocalizations evoked by noxious compression (*Pain–related behaviors*) of the left (arthritic) knee joint compared to no light (“Off”; Figure 6J, *p* < 0.005, *t* = 7.888; Figure 6K, *p* < 0.01, *t* = 4.518; *n* = 11, paired *t*-test). Just like optical silencing of BLA-CeA terminals, silencing of CeA-CRF neurons had no significant effect on mechanical withdrawal thresholds (*Pain–related behaviors*) in arthritic transgenic rats (Figure 6L, *t* = 1.729, *n* = 5, paired *t*-test).

## Discussion

Preclinical (Neugebauer et al., 2009; Apkarian et al., 2013; Thompson and Neugebauer, 2017; Neugebauer, 2020) and clinical (Kulkarni et al., 2007; Liu et al., 2010; Simons et al., 2014) studies have linked the amygdala to averse-affective aspects of pain and pain modulation. The role of individual cell types and interactions with spinal nociceptive processing as part of the descending control system remain to be determined. Evidence from preclinical research showed changes in the amygdala neurocircuitry in pain state. For instance, *in vivo* recordings revealed increased background activity and evoked responses of CeA and BLA neurons to mechanical peripheral stimuli in acute (arthritis) and neuropathic (spinal nerve ligation, SNL) pain models (Neugebauer and Li, 2003; Ji et al., 2009; Ji and Neugebauer, 2009; Ji et al., 2017; Kim et al., 2017). Mechanistic analyses in brain slice physiology experiments detected increased excitatory transmission at the PB–CeA and BLA–CeA synapses and impaired inhibitory control resulting in CeA hyperactivity in pain conditions (Neugebauer et al., 2003; Han et al., 2005; Ikeda et al., 2007; Fu and Neugebauer, 2008; Ren and Neugebauer, 2010; Kiritoshi and Neugebauer, 2018; Miyazawa et al., 2018; Wilson et al., 2019). Interestingly, amygdala pain mechanisms exhibit hemispheric lateralization with the right amygdala showing pain-related synaptic plasticity and activity changes that are involved in pain facilitatory amygdala functions (Carrasquillo and Gereau, 2008; Ji and Neugebauer, 2009; Goncalves and Dickenson, 2012; Simons et al., 2014; Miyazawa et al., 2018; Nation et al., 2018; Allen et al., 2021). Therefore, our focus on the right hemisphere in this project is justified although it will be important for future studies to perform similar manipulations in the left amygdala to determine any differences.

Pharmacological interventions that increase amygdala output have been shown to evoke pain behaviors in the absence of injury, whereas strategies that decrease amygdala output generally inhibit pain behaviors (reviewed in Neugebauer, 2020). However, there is also evidence for pain inhibitory amygdala functions (Wilson et al., 2019). A cell–type specific analysis is needed for the better understanding of amygdala pain mechanisms, and the optogenetic strategy employed here is an important step in that direction. The involvement of individual cell–types (PKCδ, SOM, CRF; see *Introduction*) in pain–related amygdala neuroplasticity has not been fully explored and is currently an area of extensive research (Wilson et al., 2019; Li and Sheets, 2020; Adke et al., 2021). Here we show that optogenetic activation of CRF neurons or of BLA input to the CeA generates nocifensive emotional responses (vocalizations) and mechanical hypersensitivity under normal conditions in the absence of tissue injury or pathology, whereas silencing of CRF neurons or of BLA input to CeA inhibits vocalizations in an arthritis pain model.

CRF output neurons in the CeA are known to project to hypothalamic nuclei, the basal forebrain and several brainstem areas involved in behaviors and pain modulation such as the periaqueductal gray (PAG), locus coeruleus (LC) and PB (Pomrenze et al., 2015; Neugebauer et al., 2020) regions. Several lines of research show that CRF projecting neurons in the CeA promote averse–affective behaviors (Fendt et al., 1997; Mccall et al., 2015; Pomrenze et al., 2015; Pomrenze et al., 2019) and that the CRF system in the amygdala is endogenously activated in pain conditions and is critically involved in amygdala pain mechanisms (Mcnally and Akil, 2002; Ji et al., 2007; Ji and Neugebauer, 2007; Fu and Neugebauer, 2008; Ji and Neugebauer, 2020; Hein et al., 2021). The BLA–CeA network is known to convey highly integrated polymodal information from thalamus and cortex and encodes averse-affective aspects of pain (Veinante et al., 2013; Corder et al., 2019; Thompson and Neugebauer, 2019; Neugebauer, 2020). The BLA neurons project to the CeA directly through excitatory synapses or indirectly via the intercalated cells (ITC) that mediate feed–forward inhibition of amygdala output from CeA neurons. ITC inhibitory tone is also driven by excitatory cortical synapses from the medial prefrontal cortex (Cheriyan et al., 2016; Kiritoshi and Neugebauer, 2018; Thompson and Neugebauer, 2019). Failure of cortical inhibitory control allows pain–related dysfunctions of this neurocircuitry resulting in increased excitatory transmission and hyperactivity of the CeA and is thought to correlate with the emotional–affective dimension of pain and pain-associated cortical deficits (Ji et al., 2010; Bushnell et al., 2013; Ren et al., 2013; Vachon-Presseau et al., 2016b; Kiritoshi and Neugebauer, 2018).

**FIGURE 7:**
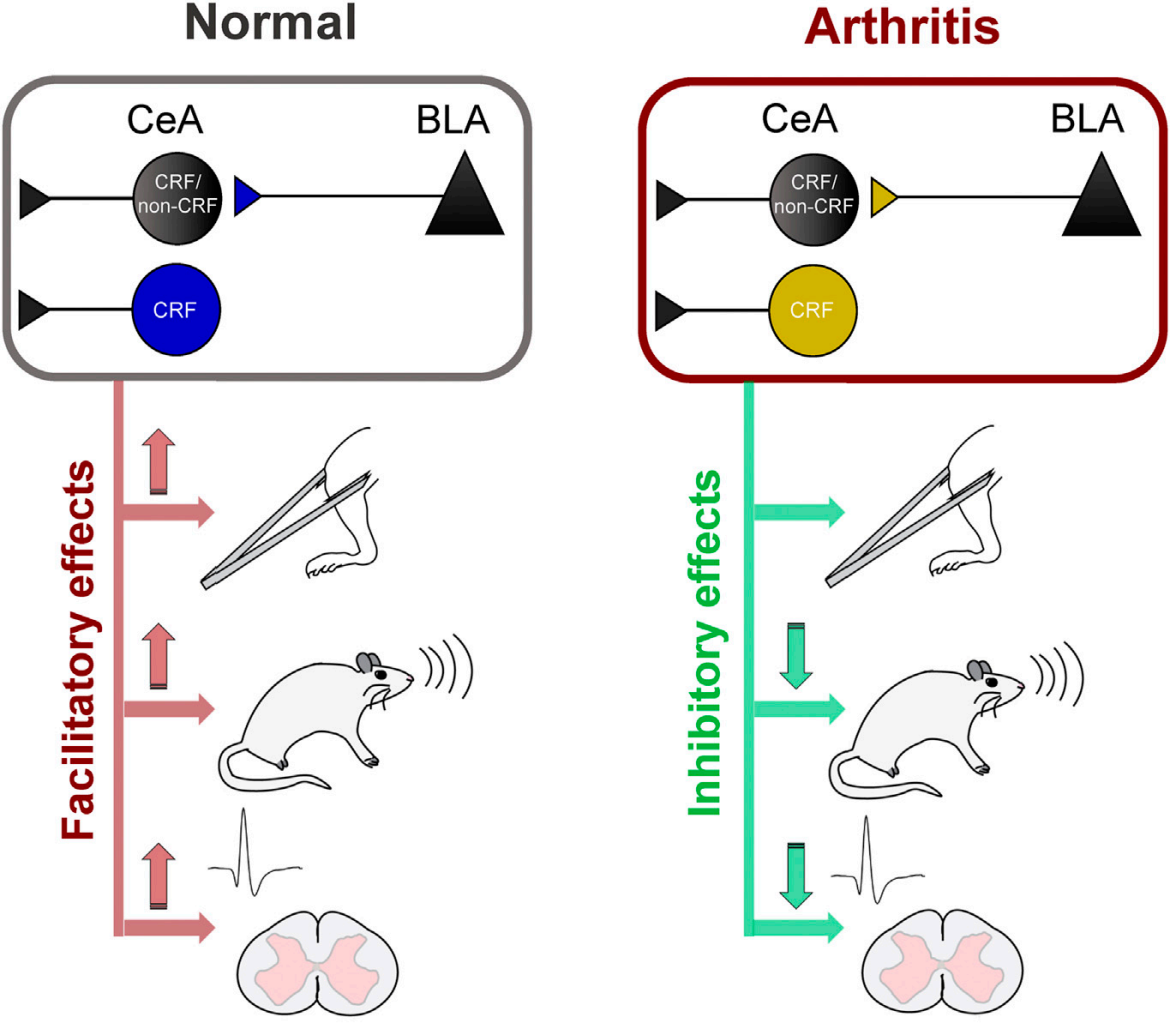
Diagram summarizing key results (Left) Activation of BLA–CeA terminals or CeA–CRF neurons has facilitatory effects on spinal nociceptive processing, vocalizations and mechanical withdrawal thresholds under normal conditions (Right) Silencing of BLA–CeA terminals or CeA–CRF neurons has inhibitory effects on spinal nociceptive processing and vocalizations, but not on withdrawal reflexes, in an arthritis pain model.

This study advances knowledge about pain-related amygdala functions by providing direct evidence for pain–facilitating effects of BLA–CeA transmission and CeA–CRF activation under normal conditions and their contribution to spinal nociceptive processing and behaviors in a pain condition (Figure 7). Sadler et al. (2017) demonstrated that optogenetic (ChR2) activation of right CeA neurons induced mechanical allodynia and increased visceromotor responses to noxious bladder distension in naïve rats, suggesting pronociceptive effects of the right amygdala to drive pain–like behaviors. Our results show that optical (ChR2) activation of glutamatergic (CaMKII) BLA–CeA terminals and of CRF–CeA neurons in the right hemisphere increases evoked responses of spinal dorsal horn WDR neurons (Figures 2, 4) and induces or increases vocalizations and mechanical sensitivity (Figure 6) under normal conditions, suggesting a critical role of these elements in so-called functional pain conditions in the absence of tissue injury or pathology. This study also showed that optical (eNpHR_3.0_) silencing of BLA–CeA terminals and CeA–CRF neurons decreased the increased activity of spinal WDR neurons (Figures 3, 5) and vocalizations (Figure 3) in the arthritis pain model, suggesting a significant contribution of amygdala activity to pain–related neuronal changes at the spinal cord level and to emotional pain-like behaviors. Optogenetic stimulation of eNpHR_3.0_ has been used before to silence CRF neurons in the amygdala (De Guglielmo et al., 2019; Ji and Neugebauer, 2020). The fact that optogenetic manipulations of the BLA–CeA and CeA–CRF elements had similar effects may suggest that excitatory BLA input to CeA targets CRF neurons or that both engage similar CeA circuits.

A surprising finding was that optical silencing of BLA–CeA and CeA–CRF neurons in the arthritis pain model did not affect mechanical hypersensitivity whereas optical activation did generate hypersensitivity. Previous studies showed that chemogenetic inhibition of PKCδ or SOM amygdala neurons had differential effects on mechanical sensitivity in a neuropathic model (1–2 weeks after induction), suggesting that they may play different, perhaps time-dependent, roles than CRF neurons (Wilson et al., 2019). Continued (15 days), but not acute, bilateral chemogenetic inhibition of CeA–CRF neurons increased the mechanical threshold in a neuropathic pain model at the 2–weeks time point, and ipsi- but not contra-lateral (to nerve injury) ablation of CeA neurons projecting to locus coeruleus also increased mechanical threshold 2 weeks after induction of neuropathic pain (Andreoli et al., 2017; Wilson et al., 2019). We interpret our data to suggest that activation of these amygdala elements has the potential to modulate not only affective but also sensory pain–like behaviors (reflexes). However, in the arthritis pain condition, these amygdala elements may not be the only or main contributors, and other cell types, synapses or pathways would need to be silenced to inhibit mechanical hypersensitivity. Still, optogenetic silencing of BLA–CeA terminals and CeA–CRF neurons inhibited the responses of spinal WDR neurons in the pain condition but not the mechanical reflex thresholds. The tests for the evaluation of the mechanical sensitivity engage a reflex arc in the spinal cord that mediates fast responses to an external stimulus (Burke et al., 1979; Brown and Fyffe, 1981). The reflexes do not necessarily require integrative brain processing, although the sensory inputs ascend in the spinal cord to reach CNS regions and spinal reflexes are modulated and under the control of supraspinal systems. It is possible that the spinal WDR neurons recorded here in the deep dorsal horn are not, or not critically, involved in the spinally mediated nocifensive reflexes even though they are targeted by projections from brainstem regions of the descending pain modulatory system recruited by higher brain regions, including the amygdala (Bingel and Tracey, 2008; Ossipov, 2012; Bushnell et al., 2013; Chen and Heinricher, 2019; Neugebauer, 2020). Perhaps neurons other than those modulated by the amygdala are more important for spinal nocifensive reflexes, and the spinal neurons modulated by the amygdala serve to convey information to the brain rather than drive motor responses, at least in pain conditions and in the arthritis model studied here. We did not determine if the spinal neurons recorded here are projection neurons. This potential shortcoming of our study remains to be addressed.

Some other caveats need to be considered. In this study two strains of rats were used to address our hypothesis. SD rats were used previously in our lab and we elected this strain for the optogenetic manipulation of the BLA–CeA synapse for consistency. Transgenic Wistar rats allowed the optogenetic modulation of CRF neurons in the CeA considering that the Cre technology was available only on the Wistar background rats at the beginning of this project (Pomrenze et al., 2015). The effects of optogenetic BLA–CeA or CeA–CRF modulations were similar arguing against significant differences in amygdala circuitry in these strains. Previous studies from our group (Ji and Neugebauer, 2020; Hein et al., 2021) and others (De Guglielmo et al., 2019) successfully used the pulsed optical protocol to manipulate the activity of specific pathways. Patch-clamp analysis validated the optogenetic protocol parameters used in the *in vivo* studies (Supplementary Figures S1, S2). In control experiments for optogenetic manipulation of the BLA–CeA synapse, we injected CaMKII–eYFP control vector (not coding opsins) into the BLA of SD rats in combination with either blue (473 nm) or yellow (590 nm) light illumination, which had no effects on spinal cord neuronal activity and behaviors. We used wild type rats injected with hChR2–eYFP expressing viral vector into the CeA in combination with blue (473 nm) light to control for optogenetic manipulation of CeA–CRF neurons in Crh–Cre transgenic rats. We decided not to test the effects of eNpHR_3.0_–eYFP expressing viral vector injected into the CeA in combination with yellow (590 nm) light in wild type rats on the WDR responses and behaviors, because we did not expect any electrophysiological and behavioral significant changes. The critical contribution of PB inputs to the CeA has been explored previously, but the selective optical modulation of PB axon terminals onto CRF and non–CRF neurons in the CeA was not addressed in this study but needs to be explored to advance our knowledge about cell- and synapse-specific roles of the PB–CeA circuitry in amygdala pain–mechanisms.

In conclusion, this study provides strong evidence for pain–facilitating effects of BLA–CeA transmission and CeA–CRF neurons on spinal nociceptive processing and pain–like behaviors under normal conditions and their critical involvement in behavioral changes and spinal nociceptive processing in an arthritis pain model (Figure 7). The data also suggest that interventions controlling amygdala activity and output may represent a desirable strategy for pain management.

## Materials and Methods

### Animals

Male Sprague–Dawley (SD) and hemizygous transgenic and wild type Crh–Cre rats (Crh = CRF, corticotropin releasing hormone/factor) on Wistar background (Pomrenze et al., 2015; De Guglielmo et al., 2019; Pomrenze et al., 2019) (initial breeding pairs kindly provided by Dr Robert Messing, UT Austin), 250–350 g at time of testing, were housed in a temperature–controlled room under a 12 h day/night cycle with unrestricted access to food and water. Experimental procedures were approved by the Institutional Animal Care and Use Committee (IACUC; protocol #14006) at Texas Tech University Health Sciences Center and conform to the guidelines of the International Association for the Study of Pain (IASP) and National Institutes of Health (NIH). Rats were randomly assigned to the different experimental groups. Experiments were performed in a blinded fashion as much as possible so that the investigators were blinded to viral vector injections, but not pain model, and different investigators performed the behavioral tests and analyses whereas electrophysiological experiments and analyses were done by the same experimenter for technical reasons.

### Experimental Protocol

Extracellular single–unit recordings of dorsal horn WDR neurons (*In vivo spinal cord electrophysiology*) or behavioral tests (*Pain–related behaviors*) were done before, during and after light application into the amygdala in naïve and arthritic rats (5–6 h after the induction, *Arthritis pain model*). For optogenetic activation or silencing of presumed glutamatergic BLA axon terminals in the CeA (*Optogenetic strategy*), a viral vector (AAV) encoding ChR2 or eNpHR_3.0_ under the control of the CaMKII promoter was injected stereotaxically into the right BLA of SD rats. For optogenetic activation or silencing of CRF neurons, a Cre–inducible viral vector (DIO–AAV) encoding ChR2 or eNpHR_3.0_ was injected into the CeA of transgenic Wistar rats. Viral vector injections were performed four weeks before the experiments to allow the expression of the light–sensitive channels. For wireless optical stimulation of ChR2 or eNpHR_3.0_ expressing CRF–CeA neurons or BLA–CeA axon terminals, an LED optic fiber was inserted into the CeA two days before the experiment. The effects of optogenetic stimulation on behavioral and electrophysiological outcome measures were assessed during 5–10 min of pulsed light application into the amygdala (CeA).

### Arthritis Pain Model

The well–established monoarthritis pain model that mimics the acute phase of the human osteoarthritis condition was induced in the left knee as described in detail previously (Neugebauer et al., 2007). Rats were briefly anesthetized with isoflurane (2–3%; precision vaporizer, Harvard Apparatus) for the separate injections of kaolin (4% in sterile saline, 100 µL) and carrageenan (2% in sterile saline, 100 µL) into the joint cavity (K/C arthritis model) followed by repetitive flexions and extensions of the leg for 5 min after each injection. This well–established paradigm produces an aseptic use–dependent mono–arthritis with damage to the cartilage, and localized inflammation in only one knee joint. K/C arthritis develops rapidly within hours and persists for more than a week, and it is associated with pain behaviors and neural activity changes in the peripheral and central nervous system. As a control group we used naïve rats undergoing similar handling but without intraarticular injections, because data from our previous studies found no differences between naïve and sham (saline injection or needle insertion) rats, justifying the use of naïve rats as an appropriate control for the K/C pain model, which is well established in our laboratories (Neugebauer et al., 2003; Gregoire and Neugebauer, 2013; Kiritoshi and Neugebauer, 2015; Mazzitelli and Neugebauer, 2019).

### Optogenetic Strategy

For optical activation or silencing of presumed glutamatergic BLA axon terminals, a viral vector (rAAV5–CaMKII–hChR2(H134R)–eYFP or rAAV5–CaMKII–eNpHR_3.0_–eYFP) (1µL, 10^12^ units/100 µL) was injected into the BLA using a 5 µL Hamilton syringe (33 gauge) to express ChR2 or eNpHR_3.0_ in BLA terminals of SD rats. For control experiments, rAAV5–CaMKII–eYFP was used. The coordinates for the injections into BLA were as follows: 2.3 mm caudal to bregma, 4.5–4.8 mm lateral to midline, and 8.0–8.5 mm deep. For optical activation or silencing of CRF neurons, a viral vector (rAAV5/EF1a–DIO–hChR2–eYFP or rAAV5/EF1a–DIO–eNpHR_3.0_–eYFP; 1 μL, 10^12^ units/100 µL) was injected into the CeA using a 5 µL Hamilton syringe to express ChR2 or eNpHR_3.0_ in CRF neurons of transgenic Wistar rats. For control experiments, viral vectors were injected in wild type rats. The coordinates for the injections into CeA were as follows: 2.5 mm caudal to bregma, 4.0–4.3 mm lateral to midline, and 7.3–7.6 mm deep. After injection, we waited 10 min for the virus to diffuse into the tissue before retracting the injection needle. All viral vectors were purchased from the vector core facility at the University of North Carolina, Chapel Hill, NC, aliquoted upon arrival, stored at –80°C and thawed before use. To activate light sensitive molecules with blue (473 nm) or yellow (590 nm) light, we used a head-mounted wireless system delivering LED light pulses (20 Hz, 5 mW, 5–10 min; Teleopto, Amuza, San Diego, CA, United States) through an optical fiber (200 μm diameter) stereotaxically implanted into the CeA two days before testing, using a small drill hole made in the anesthetized rat (isoflurane, 2–3%, precision vaporizer). Implanted fibers were held in place with dental acrylic as previously described (Thompson et al., 2015; Kiritoshi et al., 2016; Kim et al., 2017; Mazzitelli and Neugebauer, 2019; Ji and Neugebauer, 2020). Prior to surgery, function of each optic fiber was tested. The optical stimulation started 5 min before the evaluation of the electrophysiological and behavioral responses and continued during testing period (5–10 min). Each test stimulus (*In vivo spinal cord electrophysiology* and *In vivo spinal cord electrophysiology*) was applied only once.

### *In vivo* Spinal Cord Electrophysiology

Extracellular single-unit recordings were made from spinal (L2–L4) wide dynamic range (WDR) neurons, which are known to respond more strongly to stimuli of noxious than innocuous intensities (D'mello and Dickenson, 2008; Willis and Coggeshall, 2012) as described previously (Pernia-Andrade et al., 2009; Di Cesare Mannelli et al., 2015; Mazzitelli and Neugebauer, 2019; Ji and Neugebauer, 2020). To characterize neurons and for test stimuli, we used touch applied with a painter’s brush to the skin around the knee (10 s, 1 stroke per second) and innocuous (500 g/30 mm^2^) and noxious (1500 g/30 mm^2^) compression of the knee for 10 s with a calibrated forceps equipped with a force transducer whose output was displayed in grams on an LED screen. On the day of the experiment, the rat was anesthetized with isoflurane (2–3%, precision vaporizer). The spinal segments L2–L4 were exposed by laminectomy. The animal was then secured in a stereotaxic frame (David Kopf Instruments), supported by clamps attached to the vertebral processes on both sides, and the dura was carefully removed. The exposed area of the spinal cord was first framed by agar and then filled with mineral oil. Body temperature was maintained at 37°C by using a temperature–controlled blanket system. A glass insulated carbon filament electrode (4–6 MΩ) was inserted perpendicularly to the spinal cord surface using a microdrive (David Kopf Instruments) to record the activity of dorsal horn neurons. Anesthesia was maintained with isoflurane (2%, precision vaporizer) throughout the experiment. The recorded signals were amplified, band–pass filtered (300 Hz–3 kHz), and processed by a data acquisition interface (CED 1401 Plus). Spike2 software (version 4; CED) was used for spike sorting, data storage, and analysis of single–unit activity. Spike (action potential) size and configuration were monitored continuously. After a neuron was identified, a template was created for the spikes of each individual neuron during an initial recording period of 5 min, capturing the waveform within set limits of variability for parameters such as amplitude, duration, and rise time using Spike2 software. Subsequent spikes of the neuron were matched to that template (spike sorting), and only spikes within the set limits of variability were counted as signals of that particular neuron. Only neurons were included in the study whose spike configuration matched the preset template and could be clearly discriminated from background noise throughout the experiment. Only neurons were included that were identified within a depth of 1200 µm from the dorsal surface of the spinal cord, had a receptive field on the ipsilateral knee and responded more strongly to noxious (1500 g/30 mm^2^) than innocuous (500 g/30 mm^2^) compression of the knee or brushing the skin. Mechanical test stimuli were applied to the left knee joint for 10 s, and the interval between stimuli was 30 s. Neuronal activity was measured as spikes/s. Measurements were repeated about every 5 min before, during (5–10 min), and after light application into the CeA. Neuronal activity was then analyzed off-line. Net evoked activity was calculated by subtracting any ongoing activity preceding the mechanical stimulus from the total activity during stimulation.

### Pain–Related Behaviors

Vocalizations were measured before, during and after light application into CeA (*Experimental protocol* and *In vivo spinal cord electrophysiology*). Duration of vocalizations in the audible (20 Hz–16 kHz, supraspinally organized nocifensive responses) and ultrasonic (25 ± 4 kHz, limbic–driven negative emotional–affective signals) ranges (Brudzynski, 2007) were measured in naïve and arthritic rats, 5–6 h after the induction as in our previous studies (*Arthritis pain model*) (Neugebauer et al., 2007; Thompson et al., 2015; Kiritoshi et al., 2016; Mazzitelli and Neugebauer, 2019). Rats were briefly anesthetized with isoflurane (2–3%, precision vaporizer) and placed in a custom designed recording chamber (U.S. Patent 7,213,538) to ensure a fixed distance from the sound detectors. A microphone connected to a preamplifier was used to record audible vocalizations, and a bat detector connected to a filter and amplifier (UltraVox four–channel system; Noldus Information Technology) measured ultrasonic vocalizations. After recovery from the brief anesthesia, vocalizations were evoked by brief (10 s) noxious (1500 g/30 mm^2^) stimuli applied to the left (normal or arthritic) knee joint using a calibrated forceps (*In vivo spinal cord electrophysiology*). Vocalizations were recorded for 1 min and analyzed using Ultravox 2.0 software (Noldus Information Technology).

Hindlimb withdrawal thresholds were evaluated as described previously (Neugebauer et al., 2007). A calibrated forceps with force transducer (*In vivo spinal cord electrophysiology*) was used to compress the left knee joint with continuously increasing intensity until a withdrawal reflex was evoked. The average value from 2-3 trials was used to calculate the withdrawal threshold, which was defined as the force required for evoking a reflex response.

### Patch–Clamp Electrophysiology in Amygdala Slices

Brain slices containing the right amygdala were obtained from normal and arthritic SD rats and Crh-Cre Wistar rats as previously described (Kiritoshi and Neugebauer, 2015; Kiritoshi et al., 2016; Kiritoshi and Neugebauer, 2018; Hein et al., 2021). Brains were quickly removed and immersed in an oxygenated ice–cold sucrose–based physiological solution containing (in mM): 87 NaCl, 75 sucrose, 25 glucose, 5 KCl, 21 MgCl_2_, 0.5 CaCl_2_ and 1.25 NaH_2_PO_4_). Coronal brain slices (400 μm) were obtained using a Vibratome (Series 1000 Plus, The Vibratome Co., St. Louis, MO) and incubated in oxygenated artificial cerebrospinal fluid (ACSF, in mM: 117 NaCl, 4.7 KCl, 1.2 NaH_2_PO_4_, 2.5 CaCl_2_, 1.2 MgCl_2_, 25 NaHCO_3_ and 11 glucose) at room temperature (21°C) for at least 1 h before patch recordings. A single brain slice was transferred to the recording chamber and submerged in ACSF (31 ± 1°C) superfusing the slice at ∼2 ml/ min. Only one or two brain slices per animal were used.

To determine the effects of optical activation or silencing of BLA axon terminals in the CeA, whole–cell patch–clamp recordings were performed from visually identified neurons in the laterocapsular (CeLC) division using infrared (IR) DIC videomicroscopy. To determine the effects of optical activation of CeA–CRF neurons, whole–cell patch-clamp recordings were made from visually identified CRF neurons in the CeL, using DIC–IR videomicroscopy and fluorescence illumination (BX51, Olympus, Waltham, MA). YFP–expressing neurons were visualized using an LED illumination system (X–Cite Xylis) and an ET–EYFP filter set (49,003, excitation: 500 ± 20 nm, ET500/20x; emission: 535 ± 30 nm, ET535/35m; Chroma Technology Corp, Bellows Falls, VT). Recording electrodes (tip resistance 5–8 MΩ) were made from borosilicate glass and filled with intracellular solution containing (in mM): 122 K–gluconate, 5 NaCl, 0.3 CaCl_2_, 2 MgCl_2_, 1 EGTA, 10 HEPES, 5 Na_2_–ATP, and 0.4 Na_3_–GTP; pH was adjusted to 7.2–7.3 with KOH and osmolarity to 280 mOsm/ kg with sucrose. On the day of recording, 0.2% biocytin was added to the intracellular solution. Data acquisition and analysis were done using a dual 4–pole Bessel filter (Warner Instr, Hamden, CT), low–noize Digidata 1322 interface (Axon Instr, Molecular Devices, Sunnyvale, CA, United States), Axoclamp–2B amplifier (Axon Instr, Molecular Devices, Sunnyvale, CA, United States), and pClamp9 software (Axon Instr.). Headstage voltage was monitored continuously on an oscilloscope to ensure precise performance of the amplifier. If series resistance (monitored with pClamp9 software) changed more than 10%, the neuron was discarded. To characterize the electroresponsive properties of recorded neurons, depolarizing current pulses (500 ms, 25 pA step) were applied.

For neuronal activation or inhibition, a continuous light (5 ms, 5–10 mW) or a train of pulses (5 ms, 20 Hz, 5–10 mW) of blue (470 nm) or yellow (585 nm) light, delivered for 5 s onto the slice through the 40x objective of the microscope (Olympus). In one set of experiments, blue light was used to activate ChR2 expressing BLA axon terminals or CRF neurons to evoke neuronal activity, and yellow light served as control. In a different set of experiments, yellow light was used to silence eNpHR_3.0_ expressing BLA fiber terminals and CRF expressing neurons, whereas blue light served as control. In this group of experiments the recorded cell was manually depolarized to induce firing (Pomrenze et al., 2015). CeA neurons are not spontaneously active at their normal resting potential in current clamp. Therefore, manual depolarization through current injection until firing threshold was reached was necessary for the induction of neuronal firing.

### Histology

*Verification of spinal recording and optical stimulation sites in the amygdala*. At the end of each experiment, the recording site in spinal dorsal horn was marked with an electrical current (500 μA, 5 s) through the recording electrode, and spinal lumbar enlargements were removed and submerged in 4% paraformaldehyde at 4°C overnight. Brain tissues containing the optical fiber tract were also removed and placed in paraformaldehyde. Tissues were then stored in 30% sucrose before they were frozen–sectioned at 30 μm. Viral vector expression (Figures 1D,E) and locations of the tips of the optical fibers (Figure 5) were examined using bright–field or confocal microscopy. Lesion/recording sites were identified by macroscopic inspection, correlated with the depth of recording indicated on the micromanipulator, and then plotted on a graph showing the depth of the electrode tip from the dorsal surface of the spinal cord (Figures 1G,H).

*Verification of recorded amygdala neurons*. To confirm the location and to visualize the recorded neurons, the recorded slices were fixed in 4% paraformaldehyde in 0.1 M phosphate buffer (PB) for 12–24 h at 4°C. Slices were then washed in phosphate buffed saline (PBS) (3 × 10 min), permeabilized in PBS containing 0.3% Triton X–100 for 60 min, and incubated with fluorescently–conjugated streptavidin (1:1000, Streptavidin, Alexa Fluor 405 conjugate, Life Technologies) for 12–24 h at 4°C. Finally, the slices were washed in PBS (3 × 10 min), mounted on slides with Vectashield mounting medium (Vector Laboratories), and imaged under a confocal microscope (FV3000, Olympus, Center Valley, PA).

### Data and Statistical Analysis

All averaged values are presented as means ± SEM. GraphPad Prism 7.0 software (Graph–Pad Software, San Diego, CA) was used for all statistical analyses. Statistical significance was accepted at the level *p* < 0.05. One–way ANOVA (repeated measures where appropriate) with Bonferroni post hoc tests were used for multiple comparisons, and paired *t*–tests were used for comparison of two sets of data that had Gaussian distribution and similar variance as indicated.

## Data Availability

The original contributions presented in the study are included in the article/Supplementary Material, further inquiries can be directed to the corresponding authors.

## Supplementary Material

FIGURE S1 | Brain slice physiology validation of optogenetic strategy for BLA–CeA modulation. **(A)** Image of a biocytin labeled (recorded) neuron (light blue) in the CeLC, viral vector–mediated YFP expression in BLA, and YFP–expressing BLA axon terminals in CeA (green, eYFP). Recordings of a CeLC neuron **(B)** in current clamp show depolarization and induction of neuronal firing when continuous or pulsed (20 Hz) blue light (5–10 mW, 5 s) was applied **(C)** to activate ChR2–YFP expressing BLA terminals in an amygdala slice from a normal rat. Neuron was manually depolarized to –44 mV. Yellow light served as control. Scale bar 200 µm. **(D, E)** Recordings of another CeLC neuron. Same display as in (B, C) but with yellow light application to eNpHR3.0 expressing BLA terminals in an amygdala slice obtained from an arthritic rat. Blue light served as control. Neuron was manually depolarized to –43 mV to generate action potential firing.

FIGURE S2 | Brain slice physiology validation of optogenetic strategy for CeA-CRF modulation. **(A)** Image of a biocytin labeled (recorded) CeA–CRF neuron (light blue) co–expressing YFP and viral vector–mediated YFP expression in CeA–CRF neurons (green, eYFP). Recordings of a YFP–expressing neuron in a brain slice from a transgenic Crh–Cre rat **(B)** in current clamp show depolarization and induction of neuronal firing when continuous or pulsed (20 Hz) blue light (5–10 mW, 5 s) was applied **(C)** to activate ChR2 expressing CeA–CRF neurons (slice obtained from a normal rat). Neuron was manually depolarized to –53 mV. Yellow light served as control. Scale bar 100 µm. **(D, E)** Recordings of another CeA–CRF neuron. Same display as in (B, C) but with yellow light application to eNpHR3.0 expressing CeA–CRF neurons in an amygdala slice obtained from an arthritic rat. Blue light served as control. Neuron was manually depolarized to generate action potential firing.

## References

[B1] Adke A. P. Khan A. Ahn H. S. Becker J. J. Wilson T. D. Valdivia S. (2021). Cell-Type Specificity of Neuronal Excitability and Morphology in the Central Amygdala. eNeuro 8. 10.1523/eneuro.0402-20.2020 PMC7877473 33188006

[B2] Allen H. N. Bobnar H. J. Kolber B. J. (2021). Left and Right Hemispheric Lateralization of the Amygdala in Pain. Prog. Neurobiol. 196, 101891. 10.1016/j.pneurobio.2020.101891 32730859 PMC7770059

[B3] Andreoli M. Marketkar T. Dimitrov E. (2017). Contribution of Amygdala CRF Neurons to Chronic Pain. Exp. Neurol. 298, 1–12. 10.1016/j.expneurol.2017.08.010 28830762 PMC5658242

[B4] Apkarian A. V. Neugebauer V. Koob G. Edwards S. Levine J. D. Ferrari L. (2013). Neural Mechanisms of Pain and Alcohol Dependence. Pharmacol. Biochem. Behav. 112, 34–41. 10.1016/j.pbb.2013.09.008 24095683 PMC4887744

[B5] Bingel U. Tracey I. (2008). Imaging CNS Modulation of Pain in Humans. Physiology 23, 371–380. 10.1152/physiol.00024.2008 19074744

[B6] Brown A. G. Fyffe R. E. (1981). Direct Observations on the Contacts Made between Ia Afferent Fibres and Alpha-Motoneurones in the Cat's Lumbosacral Spinal Cord. J. Physiol. 313, 121–140. 10.1113/jphysiol.1981.sp013654 7277213 PMC1274440

[B7] Brudzynski S. M. (2007). Ultrasonic Calls of Rats as Indicator Variables of Negative or Positive States: Acetylcholine-Dopamine Interaction and Acoustic Coding. Behav. Brain Res. 182, 261–273. 10.1016/j.bbr.2007.03.004 17467067

[B8] Burke R. E. Walmsley B. Hodgson J. A. (1979). HRP Anatomy of Group Ia Afferent Contacts on Alpha Motoneuroness. Brain Res. 160, 347–352. 10.1016/0006-8993(79)90430-x 761069

[B9] Bushnell M. C. Čeko M. Low L. A. (2013). Cognitive and Emotional Control of Pain and its Disruption in Chronic Pain. Nat. Rev. Neurosci. 14, 502–511. 10.1038/nrn3516 23719569 PMC4465351

[B10] Carrasquillo Y. Gereau R. W. T. (2008). Hemispheric Lateralization of a Molecular Signal for Pain Modulation in the Amygdala. Mol. Pain 4, 24. 10.1186/1744-8069-4-24 18573207 PMC2443116

[B11] Chen Q. Heinricher M. M. (2019). Descending Control Mechanisms and Chronic Pain. Curr. Rheumatol. Rep. 21, 13. 10.1007/s11926-019-0813-1 30830471

[B12] Cheriyan J. Kaushik M. K. Ferreira A. N. Sheets P. L. (2016). Specific Targeting of the Basolateral Amygdala to Projectionally Defined Pyramidal Neurons in Prelimbic and Infralimbic Cortex. eNeuro 3. 10.1523/eneuro.0002-16.2016 PMC4804386 27022632

[B13] Corder G. Ahanonu B. Grewe B. F. Wang D. Schnitzer M. J. Scherrer G. (2019). An Amygdalar Neural Ensemble that Encodes the Unpleasantness of Pain. Science 363, 276–281. 10.1126/science.aap8586 30655440 PMC6450685

[B14] D’mello R. Dickenson A. H. (2008). Spinal Cord Mechanisms of Pain. Br. J. Anaesth. 101, 8–16. 10.1093/bja/aen088 18417503

[B15] De Guglielmo G. Kallupi M. Pomrenze M. B. Crawford E. Simpson S. Schweitzer P. (2019). Inactivation of a CRF-dependent Amygdalofugal Pathway Reverses Addiction-like Behaviors in Alcohol-dependent Rats. Nat. Commun. 10, 1238. 10.1038/s41467-019-09183-0 30886240 PMC6423296

[B16] DeBerry J. J. Robbins M. T. Ness T. J. (2015). The Amygdala Central Nucleus Is Required for Acute Stress-Induced Bladder Hyperalgesia in a Rat Visceral Pain Model. Brain Res. 1606, 77–85. 10.1016/j.brainres.2015.01.008 25698616 PMC4388818

[B17] Deisseroth K. (2015). Optogenetics: 10 Years of Microbial Opsins in Neuroscience. Nat. Neurosci. 18, 1213–1225. 10.1038/nn.4091 26308982 PMC4790845

[B18] Di Cesare Mannelli L. Pacini A. Corti F. Boccella S. Luongo L. Esposito E. (2015). Antineuropathic Profile of N-Palmitoylethanolamine in a Rat Model of Oxaliplatin-Induced Neurotoxicity. PLoS One 10, e0128080. 10.1371/journal.pone.0128080 26039098 PMC4454493

[B19] Fendt M. Koch M. Schnitzler H.-U. (1997). Corticotropin-releasing Factor in the Caudal Pontine Reticular Nucleus Mediates the Expression of Fear-Potentiated Startle in the Rat. Eur. J. Neurosci. 9, 299–305. 10.1111/j.1460-9568.1997.tb01400.x 9058050

[B20] Fu Y. Neugebauer V. (2008). Differential Mechanisms of CRF1 and CRF2 Receptor Functions in the Amygdala in Pain-Related Synaptic Facilitation and Behavior. J. Neurosci. 28, 3861–3876. 10.1523/jneurosci.0227-08.2008 18400885 PMC2557030

[B21] Gonçalves L. Dickenson A. H. (2012). Asymmetric Time-dependent Activation of Right Central Amygdala Neurones in Rats with Peripheral Neuropathy and Pregabalin Modulation. Eur. J. Neurosci. 36, 3204–3213. 10.1111/j.1460-9568.2012.08235.x 22861166

[B22] Gregoire S. Neugebauer V. (2013). 5-HT2CR Blockade in the Amygdala Conveys Analgesic Efficacy to SSRIs in a Rat Model of Arthritis Pain. Mol. Pain 9, 41. 10.1186/1744-8069-9-41 23937887 PMC3751088

[B23] Han J. S. Li W. Neugebauer V. (2005). Critical Role of Calcitonin Gene-Related Peptide 1 Receptors in the Amygdala in Synaptic Plasticity and Pain Behavior. J. Neurosci. 25, 10717–10728. 10.1523/jneurosci.4112-05.2005 16291945 PMC6725858

[B24] Haubensak W. Kunwar P. S. Cai H. Ciocchi S. Wall N. R. Ponnusamy R. (2010). Genetic Dissection of an Amygdala Microcircuit that Gates Conditioned Fear. Nature 468, 270–276. 10.1038/nature09553 21068836 PMC3597095

[B25] Hein M. Ji G. Tidwell D. D'souza P. Kiritoshi T. Yakhnitsa V. (2021). Kappa Opioid Receptor Activation in the Amygdala Disinhibits CRF Neurons to Generate Pain-like Behaviors. Neuropharmacology 185, 108456. 10.1016/j.neuropharm.2021.108456 33444637 PMC7887082

[B26] Ikeda R. Takahashi Y. Inoue K. Kato F. (2007). NMDA Receptor-independent Synaptic Plasticity in the Central Amygdala in the Rat Model of Neuropathic Pain. Pain 127, 161–172. 10.1016/j.pain.2006.09.003 17055162

[B27] Janak P. H. Tye K. M. (2015). From Circuits to Behaviour in the Amygdala. Nature 517, 284–292. 10.1038/nature14188 25592533 PMC4565157

[B28] Ji G. Fu Y. Ruppert K. A. Neugebauer V. (2007). Pain-related Anxiety-like Behavior Requires CRF1 Receptors in the Amygdala. Mol. Pain 3, 13. 10.1186/1744-8069-3-13 17550594 PMC1891279

[B29] Ji G. Horvath C. Neugebauer V. (2009). NR2B Receptor Blockade Inhibits Pain-Related Sensitization of Amygdala Neurons. Mol. Pain 5, 21. 10.1186/1744-8069-5-21 19400952 PMC2679723

[B30] Ji G. Neugebauer V. (2007). Differential Effects of CRF1 and CRF2 Receptor Antagonists on Pain-Related Sensitization of Neurons in the Central Nucleus of the Amygdala. J. Neurophysiol. 97, 3893–3904. 10.1152/jn.00135.2007 17392412

[B31] Ji G. Neugebauer V. (2009). Hemispheric Lateralization of Pain Processing by Amygdala Neurons. J. Neurophysiol. 102, 2253–2264. 10.1152/jn.00166.2009 19625541 PMC2776996

[B32] Ji G. Neugebauer V. (2020). Kappa Opioid Receptors in the Central Amygdala Modulate Spinal Nociceptive Processing through an Action on Amygdala CRF Neurons. Mol. Brain 13, 128. 10.1186/s13041-020-00669-3 32948219 PMC7501648

[B33] Ji G. Sun H. Fu Y. Li Z. Pais-Vieira M. Galhardo V. (2010). Cognitive Impairment in Pain through Amygdala-Driven Prefrontal Cortical Deactivation. J. Neurosci. 30, 5451–5464. 10.1523/jneurosci.0225-10.2010 20392966 PMC2868074

[B34] Ji G. Zhang W. Mahimainathan L. Narasimhan M. Kiritoshi T. Fan X. (2017). 5-HT2C Receptor Knockdown in the Amygdala Inhibits Neuropathic-Pain-Related Plasticity and Behaviors. J. Neurosci. 37, 1378–1393. 10.1523/jneurosci.2468-16.2016 28011743 PMC5299563

[B35] Kato F. Sugimura Y. K. Takahashi Y. (2018). Pain-Associated Neural Plasticity in the Parabrachial to Central Amygdala Circuit : Pain Changes the Brain, and the Brain Changes the Pain. Adv. Exp. Med. Biol. 1099, 157–166. 10.1007/978-981-13-1756-9_14 30306523

[B36] Kim H. Thompson J. Ji G. Ganapathy V. Neugebauer V. (2017). Monomethyl Fumarate Inhibits Pain Behaviors and Amygdala Activity in a Rat Arthritis Model. Pain 158, 2376–2385. 10.1097/j.pain.0000000000001042 28832396 PMC5680104

[B37] Kiritoshi T. Ji G. Neugebauer V. (2016). Rescue of Impaired mGluR5-Driven Endocannabinoid Signaling Restores Prefrontal Cortical Output to Inhibit Pain in Arthritic Rats. J. Neurosci. 36, 837–850. 10.1523/jneurosci.4047-15.2016 26791214 PMC4719019

[B38] Kiritoshi T. Neugebauer V. (2015). Group II mGluRs Modulate Baseline and Arthritis Pain-Related Synaptic Transmission in the Rat Medial Prefrontal Cortex. Neuropharmacology 95, 388–394. 10.1016/j.neuropharm.2015.04.003 25912637 PMC4466036

[B39] Kiritoshi T. Neugebauer V. (2018). Pathway-Specific Alterations of Cortico-Amygdala Transmission in an Arthritis Pain Model. ACS Chem. Neurosci. 9, 2252–2261. 10.1021/acschemneuro.8b00022 29630339 PMC6146017

[B40] Kulkarni B. Bentley D. E. Elliott R. Julyan P. J. Boger E. Watson A. (2007). Arthritic Pain Is Processed in Brain Areas Concerned with Emotions and Fear. Arthritis Rheum. 56, 1345–1354. 10.1002/art.22460 17393440

[B41] Li H. Penzo M. A. Taniguchi H. Kopec C. D. Huang Z. J. Li B. (2013). Experience-dependent Modification of a Central Amygdala Fear Circuit. Nat. Neurosci. 16, 332–339. 10.1038/nn.3322 23354330 PMC3581751

[B42] Li J.-N. Sheets P. L. (2020). Spared Nerve Injury Differentially Alters Parabrachial Monosynaptic Excitatory Inputs to Molecularly Specific Neurons in Distinct Subregions of the Central Amygdala. Pain 161, 166–176. 10.1097/j.pain.0000000000001691 31479066 PMC6940027

[B43] Liu C. C. Ohara S. Franaszczuk P. Zagzoog N. Gallagher M. Lenz F. A. (2010). Painful Stimuli Evoke Potentials Recorded from the Medial Temporal Lobe in Humans. Neuroscience 165, 1402–1411. 10.1016/j.neuroscience.2009.11.026 19925853 PMC2815048

[B44] Mazzitelli M. Neugebauer V. (2019). Amygdala Group II mGluRs Mediate the Inhibitory Effects of Systemic Group II mGluR Activation on Behavior and Spinal Neurons in a Rat Model of Arthritis Pain. Neuropharmacology 158, 107706. 10.1016/j.neuropharm.2019.107706 31306647 PMC6745268

[B45] Mccall J. G. Al-Hasani R. Siuda E. R. Hong D. Y. Norris A. J. Ford C. P. (2015). CRH Engagement of the Locus Coeruleus Noradrenergic System Mediates Stress-Induced Anxiety. Neuron 87, 605–620. 10.1016/j.neuron.2015.07.002 26212712 PMC4529361

[B46] McCullough K. M. Morrison F. G. Hartmann J. Carlezon W. A. Jr. Ressler K. J. (2018). Quantified Coexpression Analysis of Central Amygdala Subpopulations. eNeuro 5, 10-18. 10.1523/eneuro.0010-18.2018 PMC5810038 29445764

[B47] Mcnally G. P. Akil H. (2002). Role of Corticotropin-Releasing Hormone in the Amygdala and Bed Nucleus of the Stria Terminalis in the Behavioral, Pain Modulatory, and Endocrine Consequences of Opiate Withdrawal. Neuroscience 112, 605–617. 10.1016/s0306-4522(02)00105-7 12074902

[B48] Miyazawa Y. Takahashi Y. Watabe A. M. Kato F. (2018). Predominant Synaptic Potentiation and Activation in the Right Central Amygdala Are Independent of Bilateral Parabrachial Activation in the Hemilateral Trigeminal Inflammatory Pain Model of Rats. Mol. Pain 14, 1744806918807102. 10.1177/1744806918807102 30270724 PMC6243415

[B49] Nation K. M. De Felice M. Hernandez P. I. Dodick D. W. Neugebauer V. Navratilova E. (2018). Lateralized Kappa Opioid Receptor Signaling from the Amygdala Central Nucleus Promotes Stress-Induced Functional Pain. Pain 159, 919–928. 10.1097/j.pain.0000000000001167 29369967 PMC5916844

[B50] Neugebauer V. (2020). Amygdala Physiology in Pain. Handbook Behav. Neurosci. 26, 101–113. 10.1016/b978-0-12-815134-1.00004-0 PMC8059430 33889063

[B51] Neugebauer V. Galhardo V. Maione S. Mackey S. C. (2009). Forebrain Pain Mechanisms. Brain Res. Rev. 60, 226–242. 10.1016/j.brainresrev.2008.12.014 19162070 PMC2700838

[B52] Neugebauer V. Han J. S. Adwanikar H. Fu Y. Ji G. (2007). Techniques for Assessing Knee Joint Pain in Arthritis. Mol. Pain 3, 8. 10.1186/1744-8069-3-8 17391515 PMC1851005

[B53] Neugebauer V. Li W. Bird G. C. Bhave G. Gereau R. W. (2003). Synaptic Plasticity in the Amygdala in a Model of Arthritic Pain: Differential Roles of Metabotropic Glutamate Receptors 1 and 5. J. Neurosci. 23, 52–63. 10.1523/jneurosci.23-01-00052.2003 12514201 PMC6742141

[B54] Neugebauer V. Li W. (2003). Differential Sensitization of Amygdala Neurons to Afferent Inputs in a Model of Arthritic Pain. J. Neurophysiol. 89, 716–727. 10.1152/jn.00799.2002 12574449

[B55] Neugebauer V. Mazzitelli M. Cragg B. Ji G. Navratilova E. Porreca F. (2020). Amygdala, Neuropeptides, and Chronic Pain-Related Affective Behaviors. Neuropharmacology 170, 108052. 10.1016/j.neuropharm.2020.108052 32188569 PMC7214122

[B56] Ossipov M. H. (2012). The Perception and Endogenous Modulation of Pain. Scientifica (Cairo) 2012, 561761. 10.6064/2012/561761 24278716 PMC3820628

[B57] Pernia-Andrade A. J. Kato A. Witschi R. Nyilas R. Katona I. Freund T. F. (2009). Spinal Endocannabinoids and CB1 Receptors Mediate C-Fiber-Induced Heterosynaptic Pain Sensitization. Science 325, 760–764. 10.1126/science.1171870 19661434 PMC2835775

[B58] Pomrenze M. B. Giovanetti S. M. Maiya R. Gordon A. G. Kreeger L. J. Messing R. O. (2019). Dissecting the Roles of GABA and Neuropeptides from Rat Central Amygdala CRF Neurons in Anxiety and Fear Learning. Cell Rep 29, 13e14–21. 10.1016/j.celrep.2019.08.083 31577943 PMC6879108

[B59] Pomrenze M. B. Millan E. Z. Hopf F. W. Keiflin R. Maiya R. Blasio A. (2015). A Transgenic Rat for Investigating the Anatomy and Function of Corticotrophin Releasing Factor Circuits. Front. Neurosci. 9, 487. 10.3389/fnins.2015.00487 26733798 PMC4689854

[B60] Ren W. Kiritoshi T. Grégoire S. Ji G. Guerrini R. Calo G. (2013). Neuropeptide S: a Novel Regulator of Pain-Related Amygdala Plasticity and Behaviors. J. Neurophysiol. 110, 1765–1781. 10.1152/jn.00874.2012 23883857 PMC3798934

[B61] Ren W. Neugebauer V. (2010). Pain-related Increase of Excitatory Transmission and Decrease of Inhibitory Transmission in the Central Nucleus of the Amygdala Are Mediated by mGluR1. Mol. Pain 6, 93. 10.1186/1744-8069-6-93 21162731 PMC3016348

[B62] Sadler K. E. Mcquaid N. A. Cox A. C. Behun M. N. Trouten A. M. Kolber B. J. (2017). Divergent Functions of the Left and Right Central Amygdala in Visceral Nociception. Pain 158, 747–759. 10.1097/j.pain.0000000000000830 28225716 PMC5655992

[B63] Simons L. E. Moulton E. A. Linnman C. Carpino E. Becerra L. Borsook D. (2014). The Human Amygdala and Pain: Evidence from Neuroimaging. Hum. Brain Mapp. 35, 527–538. 10.1002/hbm.22199 23097300 PMC3920543

[B64] Thompson J. M. Ji G. Neugebauer V. (2015). Small-conductance Calcium-Activated Potassium (SK) Channels in the Amygdala Mediate Pain-Inhibiting Effects of Clinically Available Riluzole in a Rat Model of Arthritis Pain. Mol. Pain 11, 51. 10.1186/s12990-015-0055-9 26311432 PMC4551697

[B65] Thompson J. M. Neugebauer V. (2017). Amygdala Plasticity and Pain. Pain Res. Manag. 2017, 8296501. 10.1155/2017/8296501 29302197 PMC5742506

[B66] Thompson J. M. Neugebauer V. (2019). Cortico-limbic Pain Mechanisms. Neurosci. Lett. 702, 15–23. 10.1016/j.neulet.2018.11.037 30503916 PMC6520155

[B67] Toyoda H. Li X. Y. Wu L. J. Zhao M. G. Descalzi G. Chen T. (2011). Interplay of Amygdala and Cingulate Plasticity in Emotional Fear. Neural Plast. 2011, 813749. 10.1155/2011/813749 21912749 PMC3168900

[B68] Vachon-Presseau E. Centeno M. V. Ren W. Berger S. E. Tétreault P. Ghantous M. (2016a). The Emotional Brain as a Predictor and Amplifier of Chronic Pain. J. Dent Res. 95, 605–612. 10.1177/0022034516638027 26965423 PMC4924545

[B69] Vachon-Presseau E. Tétreault P. Petre B. Huang L. Berger S. E. Torbey S. (2016b). Corticolimbic Anatomical Characteristics Predetermine Risk for Chronic Pain. Brain 139, 1958–1970. 10.1093/brain/aww100 27190016 PMC4939699

[B70] Veinante P. Yalcin I. Barrot M. (2013). The Amygdala between Sensation and Affect: a Role in Pain. J. Mol. Psychiatry 1, 9. 10.1186/2049-9256-1-9 25408902 PMC4223879

[B71] Willis W. D. Jr Coggeshall R. E. (2012). Sensory Mechanisms of the Spinal Cord: Volume 1 Primary Afferent Neurons and the Spinal Dorsal Horn(Berlin: Springer).

[B72] Wilson T. D. Valdivia S. Khan A. Ahn H.-S. Adke A. P. Martinez Gonzalez S. (2019). Dual and Opposing Functions of the Central Amygdala in the Modulation of Pain. Cel. Rep. 29, 332–346. 10.1016/j.celrep.2019.09.011 PMC6816228 31597095

