# Supplementary figures and images for: Optogenetic Manipulations of Amygdala Neurons Modulate Spinal Nociceptive Processing and Behavior Under Normal Conditions and in an Arthritis Pain Model
Source: Front Pharmacol. 2021 May 25;12:668337. doi: 10.3389/fphar.2021.668337 (PMC8185300; doi:10.3389/fphar.2021.668337)

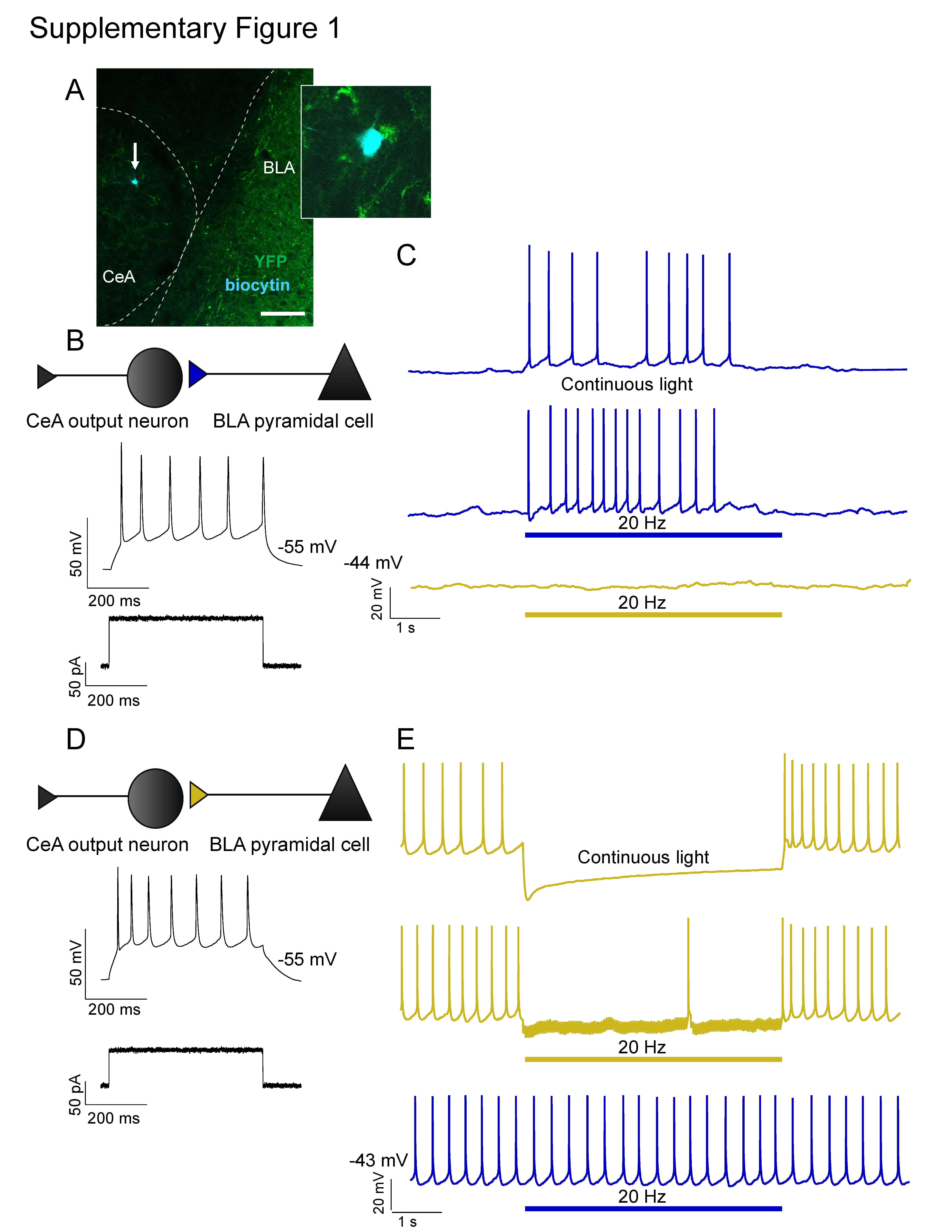

Supplement: Supplementary file 1 [file Image1.tiff]

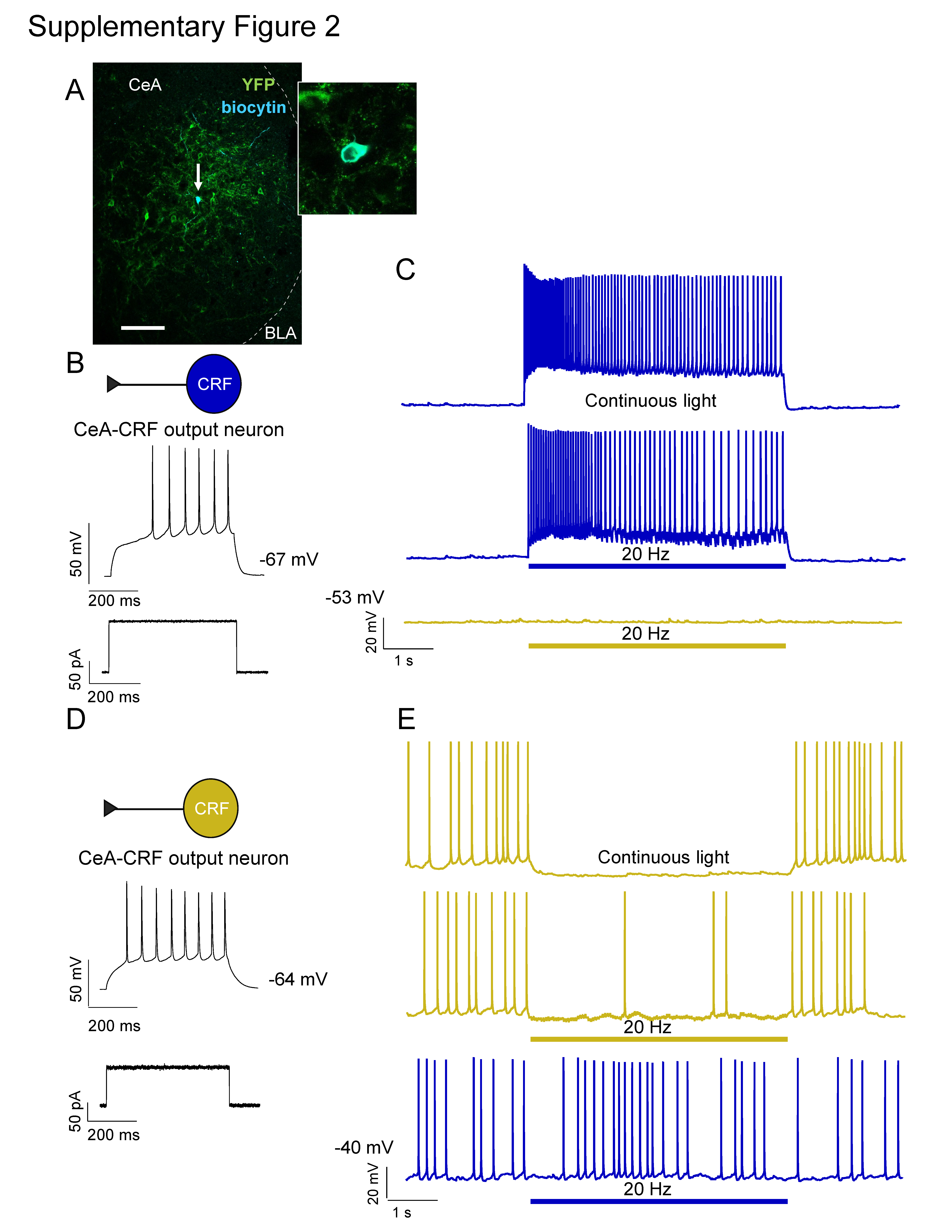

Supplement: Supplementary file 2 [file Image2.tiff]
